# Second Cancers in Classical Hodgkin Lymphoma and Diffuse Large B-Cell Lymphoma: A Systematic Review by the Fondazione Italiana Linfomi

**DOI:** 10.3390/cancers14030519

**Published:** 2022-01-20

**Authors:** Luca Nassi, Vitaliana De Sanctis, Giacomo Loseto, Chiara Gerardi, Eleonora Allocati, Sabino Ciavarella, Carla Minoia, Attilio Guarini, Alessia Bari

**Affiliations:** 1 Department of Hematology, Careggi Hospital and University of Florence, 50134 Florence, Italy; 2 Department of Radiation Oncology, Faculty of Medicina e Psicologia, Sant’Andrea Hospital, University of Rome “La Sapienza”, 00185 Rome, Italy; vitaliana.desanctis@uniroma1.it; 3 Hematology Unit—IRCCS Istituto Tumori “Giovanni Paolo II”, 70124 Bari, Italy; g.loseto@oncologico.bari.it (G.L.); s.ciavarella@oncologico.bari.it (S.C.); carlaminoia@libero.it (C.M.); attilioguarini@oncologico.bari.it (A.G.); 4 Istituto di Ricerche Farmacologiche “Mario Negri” IRCCS, 20156 Milan, Italy; chiara.gerardi@marionegri.it (C.G.); eleonora.allocati@marionegri.it (E.A.); 5 Dipartimento di Scienze Mediche e Chirurgiche Materno-Infantili e dell’Adulto, Universita’ di Modena e Reggio Emilia, 41124 Modena, Italy; alessia.bari@unimore.it

**Keywords:** survivors, classical Hodgkin lymphoma, diffuse large B-cell lymphoma, second malignancies/cancers, incidence, screening, early diagnosis, systematic review

## Abstract

**Simple Summary:**

With the presented study, the FIL researchers aimed to fill a gap in the literature regarding long-lived lymphoma patients, at least 5 years after lymphoma. These patients can develop a series of late sequelae that affect their quality of life and overall survival, in particular cardiotoxicity and secondary malignancies. This systematic review conducted by FIL researchers aimed to understand the incidence of second malignancies, consider the impact of novel therapies, and examine the best follow-up policies for their early detection. On the basis of the evidence, individualized primary risk prevention strategies are suggested, depending on the dose and volume of radiation, chemotherapy, age at treatment, and predisposing factors. When evidence was either lacking or not definitive, expert opinion was used to identify a screening schedule.

**Abstract:**

Background: The increase of lymphoma patient survival led to a modification of the incidence of long-term sequelae, including second malignancies (SM). Several groups have dealt with the incidence of SM, according to the primary treatment; however, a standardized approach for the early detection and screening of SM in the population of lymphoma survivors should be implemented. Methods: A systematic review was conducted by Fondazione Italiana Linfomi (FIL), in order to define the incidence of SM, the impact of modern radiotherapy on SM risk, and the usefulness of tailored follow-up and screening strategies for early diagnosis of SM. Classical Hodgkin lymphoma (cHL) and diffuse large B-cell lymphoma (DLBCL) survivors were investigated. The MEDLINE, Embase, and Cochrane Library databases were checked for relevant reports published up to January 2020. The selection process was reported according to the preferred reporting items for systematic reviews and meta-analyses (PRISMA) guidelines. Results: A total of 27 full-text manuscripts resulted as eligible for the analysis. The incidence of SM in cHL patients treated with ABVD was higher compared to the general population and was even higher in patients treated with intensified regimens. The risk increased over time, as well as after 10–15 years from therapy, and was augmented by radiotherapy exposure. In DLBCL, more intensive regimens (i.e., R-CHOEP or R-MegaCHOEP) vs. R-CHOP were associated with a higher SM incidence. Salvage chemotherapy and autologous stem cell transplants increased the risk of SM in both cHL and DLBCL cohorts. A lower incidence of SM, particularly of breast cancer (BC), was shown in cohorts of cHL survivors treated with reduced radiation volumes and doses (involved fields vs. extended fields), but robust trials are still lacking. Considering the advantage of a structured screening for early detection of SM, all the included studies regarded cHL survivors and screening strategy for early BC detection. Moreover, the authors discuss additional papers, to guide the early diagnosis of lung, colorectal, skin, and thyroid cancer in patients at risk due to family history, drug or RT exposure, or unhealthy lifestyles. These screening strategies all passed through patient awareness. Conclusion: A modern approach to chemotherapy and radiotherapy led to a lower risk of SM, which should be confirmed over time. Early detection of secondary cancers could be achieved through a tailored screening program, according to the individual risk profile.

## 1. Introduction

In recent years, there have been many advancements in the management of hematological malignancies, which have led to higher rates of prolonged complete remission. These improvements are particularly striking in patients with lymphoma, especially in diffuse large B-cell lymphoma (DLBCL) and classical Hodgkin lymphoma (cHL), with a progressive increase in the number of patients achieving long-lasting overall survival (OS) and progression-free survival (PFS), and with roughly 60% of patients cured in DLBCL [1] and a survival rate higher than 80% in cHL [2]. Even though late complications, such as second cancers, cardiovascular, neurological, and endocrine-metabolic toxicities, have been known for many years [3], the rising number of long-term lymphoma survivors renders late toxicity knowledge necessary, in order to manage them correctly. Dedicated outpatient clinics with a multidisciplinary approach may be of great help for a tailored follow-up and the identification of late sequelae. However, a recent survey conducted in Italian hematological centers reported that this approach is not widespread, and that there is great variability in local policies. Only 39.7% of centers have a dedicated long-term lymphoma survivor outpatient program, of which 60.9% are conducted by a multidisciplinary team and not solely by a haemato-oncologist [4].

Among the late complications, second malignancies (SM) are a great concern in cancer survivors, and a clinical and psychological challenge for people already diagnosed and treated for a neoplasia. Although many factors are involved in the development of SM, such as genetic predisposition, hormonal and immunological factors, and life-style and environmental factors [5], the impact of chemotherapy (CHT) and radiotherapy (RT) is paramount. Older CHT regimens and RT techniques, in terms of field extension and dose, are associated with a higher rate of SM [6].

Progress in the management of DLBCL and cHL in the first- and second-line includes the introduction of rituximab [7], the progressive abandonment of third generation [8] and hybrid regimens [9], the acquisition of new and less toxic RT techniques [10,11], and the refinement of indications for autologous stem-cell transplantation (ASCT) [12]. This has led to more active and less toxic first-line and rescue therapies, which may have had an impact on the development of SM. Moreover, the risk of solid tumors increases steadily with increasing follow-up time, from 5 to 15 years from treatment (in particular after RT), and remains high for at least 40 years [13,14]. For this reason, the late effects of older CHT/RT approaches can now be evaluated.

Many papers and abstracts have been published or presented at medical meetings about the incidence, risk factors, prognosis, and screening for SM (i.e., breast, lung, colorectal, skin-cancer and hematological neoplasia) in cancer survivors, but there is a lack of clear indications for long-term lymphoma patient follow-up, and a lack of dedicated guidelines regarding detection and/or screening for the early diagnosis of SM in adult lymphoma survivors. Numerous screening tools and programs for the early detection of primary cancers are widely applied to the general population. In this context, lymphoma survivors represent an at risk population, who could benefit from a tailored screening program, according to the individual risk profile (family history, age, sex, type of anti-cancer treatment).

For these reasons, through a systematic review of the literature, according to the Preferred Reporting Items for Systematic reviews and Meta-analyses (PRISMA) tool, Fondazione Italiana Linfomi (FIL) researchers aimed to evaluate three questions, set as PICO (population, intervention, control, outcome), in order to assess: (i) the incidence of SM (PICO A), (ii) the impact of old and modern RT treatments on SM development (PICO B), and (iii) the efficacy of follow-up schemes for the early diagnosis/screening of SM (PICO C) in the population of long-term cHL and DLBCL survivors, which represent the prevalent population of long-term lymphoma survivors. This would help onco-hematologists to establish a follow-up program according to the individual risk profile for the long-term survivorship period.

## 2. Materials and Methods

This systematic review is part of a series of analyses exploring the management and follow up of long-term lymphoma survivors and supporting the FIL position statements [15,16,17,18]. The scope of the position statements, as well as the clinical questions and PICOs for each question, were discussed and agreed by the FIL ‘Long-term Survivor Committee’ and presented at the FIL congress in 2019. The study was not registered on Prospero as part of a larger position paper, which was composed by several papers; the position paper as a whole was presented at the International Conference on Malignant Lymphoma/European Hematology Association annual congress in 2021 [19]. We used the PRISMA guidelines to report the results [20].

### 2.1. Study Identification

MEDLINE (via PubMed), the Cochrane Library, and EMBASE were systematically searched from January 1990 to January 2020, with no language or publication type restrictions. Search terms included extensive controlled vocabulary (MeSH and EMTREE) and free-text keywords, combining the conditions (Hodgkin disease, diffuse large B-cell lymphoma), interventions (e.g., CHT and RT), and outcomes of interest (e.g., second cancer, patient surveillance, early diagnosis). Details of the search strategies can be found in the Supplementary Data S1. We checked the reference lists of relevant studies to retrieve further studies and congress abstracts and searched study registries for unpublished or ongoing studies. 

### 2.2. Eligibility Criteria

We included both primary studies (randomized controlled trials (RCTs), prospective and retrospective cohort studies, and registry studies) and systematic reviews including these study designs. Studies involving long-term (≥5 years disease or treatment-free), adults (≥18-year-old at diagnosis) cHL or DLBCL survivors were selected. The included studies assessed (i) the incidence of SM in patients treated with ABVD, BEACOPP, and R-CHOP regimens, with or without RT, and in patients treated with second-line therapies including ASCT (the administration of allogeneic transplantation was an exclusion criteria), named PICO A; (ii) the impact of new RT approaches (i.e., volume reduction, dose reduction vs. previous one) on modifying the incidence of SM, named PICO B; and (iii) the effectiveness of management and early diagnosis of SM using scheduled follow-up/monitoring/screening schemes that include laboratory and/or instrumental tests, named PICO C. Table 1 reports the clinical questions and corresponding PICOs addressed by this review.

### 2.3. Study Selection and Data Extraction

Two reviewers independently screened the title and abstract to select the studies. One reviewer reviewed the full-text publication to confirm the eligibility and extract the relevant information from the included trials. A second reviewer checked the eligibility and the data extraction, to increase the accuracy of the process. Any discrepancies were resolved by consensus and arbitration by a third author. Data collected from each study comprised the following predefined items: (1) Study identifier (first author, year of publication); (2) Reference; (3) Other publication; (4) Study design; (5) Population; (6) Study duration; (7) Follow-up; (8) Sample size; (9) Intervention/control group; (10) Outcome measure; (11) Main results; (12) Conclusion; (13) Risk of bias/quality assessment. A predefined spreadsheet (Excel 2007, Microsoft Corporation^®^, Redmont, WA, USA) was used for data extraction. Data selection and extraction are presented in Supplementary Materials (Table S1). 

### 2.4. Risk of Bias and Quality of Evidence Assessment

We assessed the methodological quality of the included systematic reviews using the AMSTAR 2 tool [21], the risk of bias for the RCTs using the Cochrane risk of bias (ROB) [22], and the quality of cohort and registry studies using the Newcastle–Ottawa scale (NOS) [23]. The risk of bias and quality of evidence assessment was carried out by one reviewer and checked by a second. (Supplementary Materials, Table S2). 

### 2.5. Data Synthesis

As we expected a substantial degree of heterogeneity among the included studies, we did not pool data in the meta-analyses. For each clinical question, the included studies providing relevant information were summarized narratively and tabulated, to highlight similarities and differences in the methods and results. We focused on the review outcomes (Table 1).

## 3. Results

### 3.1. What Is the Incidence of Second Cancers in cHL or DLBCL Long-Term Survivors after First- or Second-Line Treatments including ASCT?

**Figure 1:**
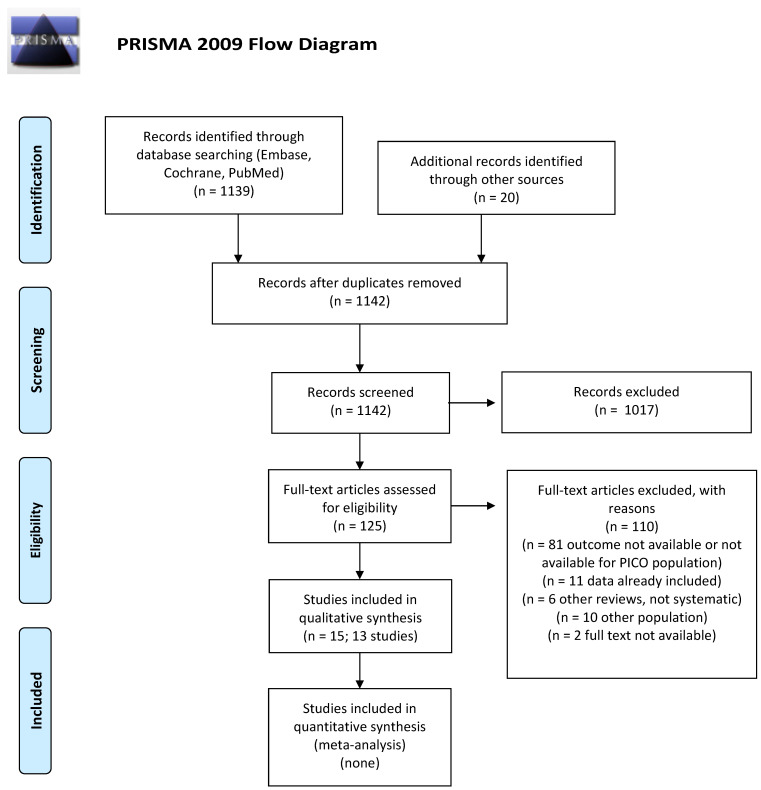
PICO A: PRISMA flow-chart for incidence of secondary cancer.

We screened 1142 abstracts and then retrieved 125 relevant publications as full texts. Of these, 110 were excluded and 15 full papers representing 13 studies were included in the final sample and relative analysis. The included studies were one systematic review, one pooled analysis of RCTs, one pooled analysis of non-randomized trials, three RCTs, and seven retrospective cohort studies. Details of the screening process, including reasons for full-text exclusion, are reported in Figure 1 (PRISMA flow-chart).

The included manuscripts are shown in the text, according to the following order: cHL survivors treated with front-line therapy, DLBCL survivors treated with front-line therapy, cHL and DLBCL survivors treated with salvage CHT and ASCT, and meta-analysis. 

The results are summarized in Table 2.

An abstract by Andre et al. [49] reported the pooled analysis of four randomized trials comparing ABVD vs. BEACOPP. Their results were recently published as a full paper [24]: 622 patients received ABVD, while the remaining 605 patients received BEACOPP (baseline or escalated according to protocol). The median follow-up was 7 years (95%; 6.6–7.2) and was similar in both arms. The incidence of SM was a secondary endpoint of the study, with OS as the primary endpoint; overall 63 SM occurred in 61 patients. The incidence of SM was 6.3% per 1000 person-years (PY) (95%; 4.29–9.39) in patients who received ABVD, and 9.6% per 1000 PY (95%; 4.29–13.25) in those who were treated with BEACOPP. A total 13 cases of secondary acute myeloid leukemia (sAML) were reported in patients who received BEACOPP, compared to none in the ABVD arm. There were 12 cases of non-Hodgkin lymphomas (NHL) (4 after BEACOPP, 8 after ABVD), and 33 secondary solid tumors (15 after ABVD, 18 after BEACOPP). The authors concluded that there was no clinically significant difference in terms of OS between ABVD and BEACOPP, and that the major cause of death after BEACOPP was SM. The authors acknowledged that the median follow-up was short, in order to analyze late toxicities, and that a longer follow-up would be necessary to correctly evaluate the impact of ABVD and BEACOPP on fertility, late toxicities, and OS. The studies included in the pooled analysis were judged to be at low risk of bias for randomization and allocation processes, detection, and reporting biases, while the risk was unclear for the attrition bias. Due to the nature of the open label trials, risk was high for blinding of participants and personnel.

Torok et al. [25] carried out a retrospective analysis of a cohort of 90 patients with unfavorable risk early stage cHL treated with ABVD and low dose RT. They observed seven SM, in particular two basal cell carcinomas, two NHL, one colon cancer, one sAML, and one small-cell lung cancer (LC). The median time to diagnosis of SM was 10 years (range: 1–14 years), but all three hematological malignancies occurred within 3 years from diagnosis. One patient had a limited stage follicular lymphoma in the left inguinal area, which was treated only with RT. The other lymphoma was a limited-stage DLBCL, which was also treated with RT alone. This patient relapsed with systemic disease and died of disease progression. The small-cell LC was observed in a smoker 13 years after treatment, and was the only malignancy that occurred within a RT field. Overall, this retrospective, cohort study was considered of intermediate quality for risk of bias due to the absence of a non-exposed cohort, no description of ascertainment of exposure, and no statement on the adequacy of follow-up.

Our systematic review identified a study by Sasse et al. [50], which was a long-term analysis of four prospective studies of the German Hodgkin Study Group (GHSG). We discarded the study and considered the full papers of the single trials. Three of them [51,52,53] were included in the meta-analysis by Franklin et al. (described later in the text) [36]. The fourth one reported the results of the GHSG HD7 randomized trial [26], which compared extended field radiation therapy (EFRT) plus involved field (IFRT) boost as standard arm with ABVD plus EFRT in 627 patients with early favorable cHL. After a median observation time of 87 months, 39 SM were observed (6.2%). There were three sAML/myelodysplastic syndrome (MDS), 21 solid tumors, 14 NHLs, and one chronic myeloid leukemia. The most frequent solid cancers included small-cell LC (5 cases), skin cancers (4 cases), and breast cancers (BC) (3 cases). Eleven of the solid tumors occurred in the irradiation fields, three in non-irradiated areas; for seven tumors the irradiated areas were unknown or unclear. There were no significant differences between treatment arms, with similar rates in each arm in the Kaplan-Meier curves for SM. The incidence was fairly constant, at approximately 0.8% per year between years 2 and 9; numbers at risk were too small for a reliable estimate (SE 3%) beyond year 9. In the long-term analysis reported by Sasse et al. [49], the 15-year estimates for any SM were 14% for EFRT plus IFRT boost and 16% for the combined modality treatment (CMT). The incidence of SM was higher in older patients (*p* < 0.0001) and in those with B symptoms at diagnosis (p. 012). This study was judged at low risk of bias for randomization and allocation processes, as well as for the detection, attrition, and reporting biases. However, being an open label trial, the risk was high for blinding of participants and personnel.

Schaapveld et al. [27] retrospectively evaluated a cohort of 3905 patients with cHL, treated between 1965 and 2000 with CHT, RT, or CMT, with a median follow-up of 19.1 years. During this period, 1055 SM were observed in 908 patients, while a third malignancy developed in 130 patients, and a fourth developed in 17. The risk of a SM was higher than in the general population (standardized incidence risk (SIR) 4.6; 95% confidence interval (CI), 4.3 to 4.9), with 121.8 excess cancers per 10,000 PY. BC represented 20.4% of the excess risk of any SM, with an overall absolute excess risk (AER) of 24.9 cases per 10,000 PY among men and women; considering the females only, the AER of BC was 54.3 cases per 10,000 PY, representing 40.5% of the excess risk of any second cancer (134.0 cases per 10,000 PY). Thyroid cancer, soft-tissue sarcoma, mesothelioma, and NHL had a risk that were more than 10-times as high as in the general population, whereas for esophageal, gastric, pancreatic cancer, and LC, as well as leukemia, SIRs were 5- to 10-times as high. Even 35 years after the treatment for cHL, the SIR for any SM remained high (SIR for ≥35 years, 3.9; 95% CI, 2.8 to 5.4), whereas the AER increased steadily over time (*p* < 0.001 for trend). SIRs did not differ in a significant way between men and women. The SIRs for solid SM decreased with increasing age at diagnosis of cHL (*p* < 0.001 for trend), rose over the first 15 years of follow-up, and remained stable thereafter. However, the decrease of SIRs with increasing age at diagnosis was not observed for LC as much as they were for BC and gastro-intestinal tract cancer. Comparing treatments within the cohort, a multivariable analysis showed that patients who received supradiaphragmatic field RT, not including the axilla, had a much lower risk of a solid SM than patients who received complete mantle-field RT (hazard ratio (HR), 0.63; 95% CI, 0.49 to 0.83), largely due to a lower risk of BC. A cumulative procarbazine dose of 4.3 g or more per square meter of body-surface area, a dose which has been associated with premature menopause, was associated with a significantly lower risk of BC (HR for the comparison with no CHT, 0.57; 95% CI, 0.39 to 0.84) but a higher risk of gastrointestinal cancer (HR 2.70; 95% CI, 1.69 to 4.30). The authors concluded that the risk of SM did not appear to be lower in the most recently treated patients (1989–2000). Overall, this retrospective cohort study was considered of good quality for risk of bias according to the Newcastle–Ottawa scale, despite the absence of a non-exposed cohort.

An abstract by Frontzek et al. [54] reported the results of a randomized, prospective German study which compared conventional R-CHOEP14 with intensified R-megaCHOEP in younger patients with high-risk DLBCL. Their results were recently published as a full paper [28]. Of the 275 enrolled patients, 262 were analyzed, of which 130 were treated with R-CHOEP14 and 132 with R-megaCHOEP. After a median follow-up of 9.3 years, 22 SM were observed (8.4%); in spite of a very high dose of etoposide (total 4 g per square meter) in the R-mega-CHOEP arm the incidence of SM was similar in both treatment arms (9% vs. 8%). There were two cases of sAML and one case of sMDS per arm. The median time to diagnosis of sMDS or sAML was 44 months, while the median time to diagnosis of a solid tumor was 72 months. This study was considered at low risk of bias for randomization and allocation processes, as well as for detection, attrition, and reporting biases. As an open label trial, the risk was high for blinding of participants and personnel.

In 2010, Coiffier et al. [29] reported long-term data from the LNH-98.5 trial comparing R-CHOP to CHOP in 399 elderly patients with DLBCL. After a median follow-up time of 10 years, 43 patients (10.8%) developed a SM, 22 in the CHOP arm and 21 in the R-CHOP arm; 28 of these patients died, 16 in the CHOP arm (death secondary to SM in 12 patients) and 12 in the R-CHOP arm (death secondary to SM in 10 patients). A third cancer appeared in three patients, one in the CHOP arm and two in the R-CHOP arm; a total of 46 SM were then observed in 43 patients after entering the study. The authors observed no pattern in the type of SM that occurred: sMDS or sAML were observed in two pts in each arm. The most frequently observed solid tumors in the CHOP and R-CHOP arms were LC (7 and 4, respectively), colon cancer (3 and 4, respectively), prostate cancer (3 and 2, respectively), BC (3 and 1, respectively), and bladder cancer (0 and 2, respectively). All other cancers (kidney, melanoma, myeloma, ovary, liver, pleura, head and neck, esophagus, skin epidermoid; a SM was of unknown origin) were present in one patient each. The authors concluded that the incidence of SM was not higher in the R-CHOP arm. This study was considered at low risk of bias for randomization and allocation processes, as well as for detection, attrition, and reporting biases. Being an open label trial, the risk was high for blinding of participants and personnel.

Castellino et al. [30] reported the results of two prospective phase-2 trials (112 pts) combining lenalidomide and R-CHOP21 in DLBCL. After a follow-up of 5.1 years, a SM was observed in seven patients (6.3%). Histology of SM was NHL in two cases, and with sAML, metastatic adenocarcinoma of unknown origin, prostate cancer, rectal adenocarcinoma, and non-melanotic skin tumor in one patient each. The median time from the end of treatment to the SM was 16.4 months (range: 5.7–53.3 months). Overall, this retrospective, cohort study was considered of intermediate quality for risk of bias due to the absence of a non-exposed cohort, the short follow-up, and no statement on the adequacy of the follow-up.

Paudel et al. [31] evaluated the role of total lymphoid irradiation associated with high-dose therapy, with carboplatin, etoposide, and cyclophosphamide as a conditioning regimen for ASCT in 89 relapsed and refractory cHL patients, with a median age at transplantation of 31 years (range 18–55 years). After a median follow-up of 5.6 years, eight patients developed SM, with five cases of hematological malignancy (5.6%) and three solid tumors (3.4%). Three of the patients with subsequent hematologic SM had received MOPP as CHT. A patient with melanoma on their back had a history of tanning bed use and disease was within the RT fields, but the other solid tumors were outside of the radiation fields. There were four deaths, all patients with hematologic SM. All the patients who developed solid SM were alive at the time of the analysis. Overall, this retrospective, cohort study was considered of intermediate quality for risk of bias, owing to the absence of a non-exposed cohort, the short follow-up exposure, and no statement on the adequacy of the follow-up.

Pingali et al. [32] reported the results of a retrospective study involving 310 patients with relapsed or refractory cHL who received ASCT with BEAM conditioning and an ifosfamide-etoposide based mobilization. After a median follow-up of 80 months (range 1–180 months), the cumulative incidence of SM was 11% in the cohort, with 13 sMDS/sAML, three basal cell carcinomas, two squamous cell cancers, one prostate cancer, one myeloproliferative neoplasm, and one sarcoma. The incidence of SM was significantly higher in patients who were older than 55 years at diagnosis (30% versus 8%; *p* < 0.001); this difference was still present after the exclusion of second skin cancers, with an incidence of 22% versus 7% in those younger than 55 years at diagnosis (*p* = 0.003). Time from initial diagnosis to transplantation (HR 1.3; 95% CI, 0.5 to 3.3; *p* = 0.50) and RT (HR 0.5; 95% CI, 0.2 to 1.4; *p* = 0.20) were not significant predictors of SM in the cohort; on multivariate analysis only age >55 years at diagnosis was a predictor of the rate of SM. MOPP, which was performed as first-line CHT in 20 patients, all of whom were <55 years of age, was associated with a higher incidence of SM (HR 6.3; *p* < 0.001 in univariate analysis) compared with ABVD. Overall, this retrospective cohort study was considered of good quality for risk of bias due to the absence of a statement on the adequacy of follow-up and despite the absence of a non-exposed cohort.

Sibon et al. [33] evaluated a population of 245 relapsed or refractory cHL treated in the H96 LYSA/SFGM-TC trial. The patients were stratified into two groups. A total of 150 patients were considered as poor-risk (refractory and unfavorable relapses) and received a tandem ASCT (first ASCT with BEAM or CBVM conditioning, followed by a second ASCT with cytarabine and melphalan associated with total body irradiation (TBI) in patients who did not receive RT) or busulphan (in patients who had already received RT). The other 95 patients considered as intermediate-risk received a single ASCT with BEAM conditioning. After a median follow-up of 10.3 years, 16 SM were reported. Overall, from inclusion, the 10- and 15-year cumulative incidences of SM were 8% and 15%, respectively. The 10-year cumulative incidence of SM in intermediate- or poor-risk patients was 15% and 1.5%, respectively. Considering only patients who did not relapse after completing ASCT (*n* = 145), the cumulative incidences of SM were 9% and 13%, at 10- and 15-years, respectively. For intermediate-risk patients (*n* = 70), the cumulative incidence of SM was 16% and 24%, while for poor-risk patients (*n* = 75) it was 2% and 2% at 10- and 15-years, respectively. The five sAML were all fatal, while some patients with NHL or solid tumors achieved long-term survival. In the discussion, the authors stressed the very different incidence of SM in the intermediate-risk subgroup, which experienced a particularly high incidence, and the poor-risk subgroup, whose incidence was surprisingly low, considering the tandem ASCT. Overall, this retrospective cohort study was considered of good quality for risk of bias, despite the absence of a non-exposed cohort.

Minn et al. [34] also evaluated the risk of SM in patients with relapsed or refractory cHL who were treated with ASCT at Stanford University and survived more than two years after the procedure. The study involved 154 patients, who received a conditioning based on the combination of etoposide and cyclophosphamide associated with TBI or carmustine or lomustine. After a median follow-up of 10.2 years, 20 SM (13%) were observed: 18 occurring at least 2 years after ASCT, and two developing within the first 2 years after ASCT (sMDS). The 5-, 10-, and 15-year cumulative incidences of developing a SM were 5%, 8%, and 12%, respectively. Thirteen of the 20 patients diagnosed with a SM had died of their second cancer at the time of the analysis; eight cases were sMDS/sAML, two LC, one each for colon cancer, gastric cancer, and NHL. MDS were diagnosed in two patients prior to 2 years, but they died 2 years after ASCT. The 10- and 15-year cumulative incidence of death from SM was 8% and 10%. Seven patients with a SM were alive at the time of analysis (three invasive ductal BC, one each for abdominal liposarcoma, thyroid cancer, endometrial stromal sarcoma, and ampullary adenocarcinoma). AER was 160 per 10,000 PY. The relative risk (RR) of SM compared with patients with cHL in the Surveillance, Epidemiology, and End Results Program (SEER, https://seer.cancer.gov (accessed on 27 April 2012)) registry was not elevated 5–10 years after ASCT, but was higher between 2 and 5 years and 10 years after ASCT. The overall RR of SM was 3.0, compared with cHL patients from SEER, the AER was 123 per 10,000 PY. The authors also compared the risk of SM in the ASCT cohort with the risk in all 1031 cHL patients who were treated at Stanford without ASCT, finding a higher risk in the ASCT cohort for the interval of 2–5 years after treatment; however, the overall risk of SM in the ASCT cohort was not elevated compared with the non-ASCT population (RR 1.5, 95% CI, 0.91–2.42). Overall, this retrospective, cohort study was considered of good quality for risk of bias due to the absence of a statement on the adequacy of follow-up, and despite the absence of a non-exposed cohort.

Tarella et al. [35] reported the incidence of SM in patients with NHL and cHL undergoing high-dose sequential therapy with ASCT. The study included 234 patients with relapsed or refractory cHL, who were considered separately. It did not consider as a separate group the 569 patients with DLBCL, who were part of a ‘high-grade’ category, which also included Burkitt lymphoma. We decided, therefore, to report the cumulative incidences of sMDS/sAML and solid SM for the cHL patients only. After a median follow-up of 6.9 years, the cumulative incidence of sMDS/sAML in cHL at 5-, 10-, and 15-years was 4.1%, 5%, and 15%, respectively, while the cumulative incidence of solid SM was 1.8, 5.5, and 6.9% respectively. Risk analysis for sMDS/sAML and solid SM were performed for the whole populations and not only for cHL patients. Compared to an age-matched population the overall SIR for sMDS/sAML was 2.6 (95% CI, 2 to 3.4), with a higher risk in patients younger than 45 years (SIR, 7.2; 95% CI, 4.5 to 11.4) and patients 45 to 65 years of age (SIR 2.1; 95% CI, 1.5 to 2.9). The SIR for solid SM was 3.2 (95% CI, 2.5 to 4.1) and the risk was higher for patients younger than 45 years (SIR, 7.6; 95% CI, 4.8 to 11.9) and patients of 45 to 65 years of age (SIR 2.7; 95% CI, 2 to 3.6). The median OS of patients with sMDS/sAML was 10 months. In contrast, patients who developed a solid tumor had a median OS of 3.8 years. Overall, this retrospective, cohort study was considered of good quality for risk of bias due to the absence of a statement on the adequacy of follow-up, despite the absence of a non-exposed cohort.

Our selection included a Cochrane review by Franklin et al. [36] that evaluated the impact on SM of the optimization of CHT and RT for cHL, by analyzing 21 eligible trials. In patients treated with CHT, the omission of additional RT could reduce the incidence of SM (Peto odds ratio (OR) 0.43, 95% CI, 0.23 to 0.82, low quality of evidence), with an estimated reduction of 8-year SM risk from 8% to 4%. The authors observed that the decrease would be particularly important for sAML. There was insufficient evidence to determine differences in OS, while a higher PFS rate was observed in patients who received CHT, with limited confidence, due to the high levels of statistical heterogeneity between studies (HR 1.31; 95% CI, 0.99 to 1.73) and a moderate quality of evidence. The authors also investigated the number of CHT courses (fewer cycles probably had little or no effect on SM risk; Peto OR 1.10; 95% CI, 0.74 to 1.62, with a moderate quality of evidence) and the role of intensified CHT in patients with advanced stage disease, with an insufficient evidence to determine the effect of CHT intensification (Peto OR 1.37; 95% CI, 0.89 to 2.10), and a low quality of evidence. In these patients the incidence of secondary acute leukemias (and for all SM in younger patients) was probably higher than among those who had treatment with standard-dose ABVD-like protocols. In contrast, the intensified CHT protocols probably improved the PFS (8-year PFS 75% versus 69% for ABVD-like treatment, HR 0.82; 95% CI, 0.7 to 0.95, moderate quality of evidence), with no conclusive evidence for improved survival with intensified CHT (HR 0.85; 95% CI, 0.70 to 1.04). Based on the AMSTAR2 tool, the quality of the meta-analysis conducted by Franklin et al. on the optimization of CHT and RT for untreated cHL patients, with respect to second malignant neoplasms, OS, and PFS, was of high quality. A full assessment of the AMSTAR tool is in the Supplementary Materials (Table S2).

### 3.2. Has the Incidence of Second Cancers in cHL or DLBCL Long-Term Survivors Who Underwent First or Second Line CHT and ASCT Changed with the Introduction of Modern RT?

**Figure 2:**
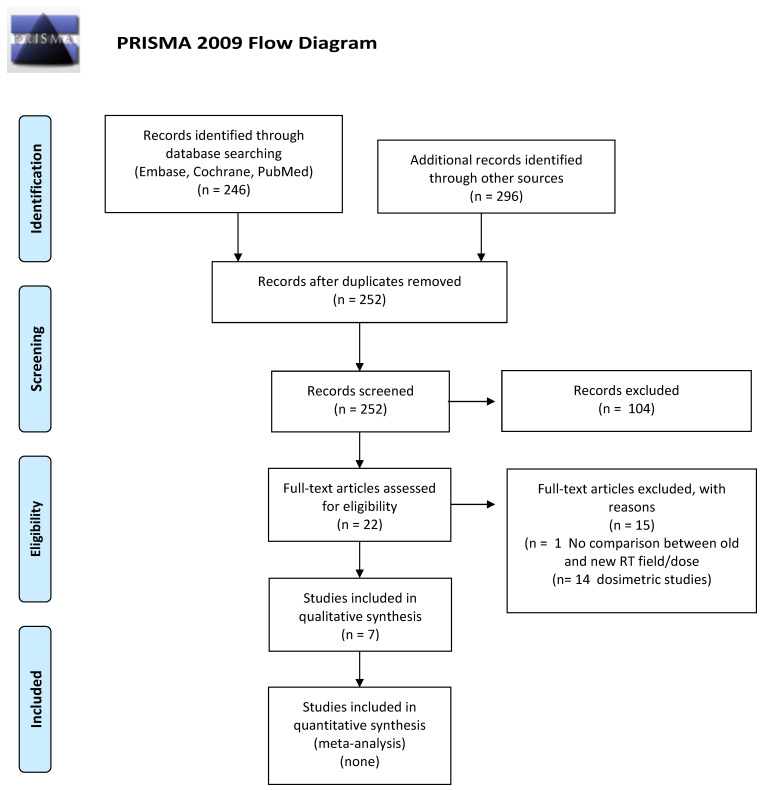
PICO B: PRISMA flow-chart for impact of modern RT on incidence of second cancer.

We screened 252 abstracts, and then 22 relevant publications were retrieved as full texts. Of these, 15 were excluded and seven studies were included in the final sample and relative analysis. Of the seven included studies, there were no RCTs, five were retrospective, and two systematic reviews. Details of the screening process, including reasons for full-text exclusion, are reported in Figure 2 (PRISMA flow-chart). The results are summarized in Table 2.

Patel et al. [37] recorded a significant reduction in all-cause mortality (also second tumors) in 1541 patients with early-stage cHL treated with RT with or without CHT in the most recent era, as compared to those treated in earlier time periods. In fact, the 15-year OS rates were 78%, 85%, and 88% (*p* = 0.0016), for patients treated during the periods 1968–1982, 1983–1992, and 1993–2007, respectively. In particular, 611 cHL patients treated from 1993 to 2007 with a median follow-up of 9.0 years received lower RT doses to smaller fields and ABVD CHT. After censoring the follow-up time at 10 years, to account for the differential follow-up between the three groups of patients, a Cox proportional hazards model was used to analyze those involved versus EFRT as a binary variable, while the RT doses were divided as follows: <2000 cGy, 2000 to <3600 cGy, or >3600 cGy. The incidence rates (IR) of competing deaths for SM were as follows: before 1983 the IR was 4.23, from 1983 to 1992 IR was 3.44, and from 1993 to 2007 IR was 2.41 for the first decade of follow-up; for the second decade, before 1983 the IR was 8.34, from 1983 to 1992 IR was 6.59, and from 1993 to 2007 IR was 4.31. When analyzed in a multivariable analysis, there was a trend toward significance for radiation field volume, only for all-cause mortality, probably due to the small number of events within each treatment period. The authors, therefore, concluded that a limited radiation field may be related to a reduction of all-cause mortality, in particular SM and cardiovascular disease, in the first decade of follow-up. 

Similar results were reported by LeMieux et al. [38] in a series of 8807 early stage cHL patients treated from 1988 to 2009, with a median follow-up of 7.2 years. When analyzed on multivariate analysis, patients treated between 2000 and 2009 were associated with a HR for SM of 0.77, compared to those treated between 1988 and 1999 (*p* = 0.02). Although no specific information regarding the radiation volume and/or dose was available, the authors identified 1999 as the time when physicians began to use IFRT instead of EFRT in cHL patients. 

Although Schaapveld et al. [27] showed that the risk of second solid cancers did not reduce in 3905 cHL patients treated between 1989–2000 (median follow-up of 19.1 years) compared to those treated in an earlier period, the risk of BC was lower when the radiation volume did not include the axilla (HR = 0.37; 95% CI, 0.19 to 0.72). These studies, while recording a positive trend of a lower incidence of second tumors after the introduction of a smaller radiation volume, such as IFRT, suffered from a lack of specific volumetric and dosimetric data regarding the RT treatment actually delivered and the volume of the organs at risk (OAR) irradiated. Moreover, patients under the age of 18 were also enrolled. 

The first study to specifically assess whether sparing the volume of breast tissue using a smaller radiation volume was related to a decreased risk of second BC was published by De Bruin et al. [39]. They analyzed 1122 female cHL patients, treated from 1965 to 1995 with CHT and/or different RT volumes (mantle field or IFRT). After a median follow-up of 17.8 years, the radiation volume involving the axillary, mediastinal, and neck nodes (mantle field) was associated with a 2.7-fold increased risk of BC (95% CI, 1.1 to 6.9) compared with similarly dosed (36 to 44 Gy) mediastinal irradiation alone (mainly IFRT). Patients under the age of 18 were also enrolled.

These data were confirmed by Conway et al. [40] in 734 cHL women, followed for more than 10 years. The 20-year estimated risk for second BC was 7.5% for mantle field, 3.1% for smaller RT (IFRT and more recently involved site RT (ISRT) and involved nodal RT (INRT)), and 2.2% for CHT only. In conclusion, mantle RT was associated with a higher risk of second BC compared to CHT only (HR = 2.9; 95% CI, 1.4–6.0; *p* = 0.004) and to smaller radiation volume (HR = 3.3; 95% CI 1.3–8.4; *p* = 0.01). In particular, the modern RT approaches based on smaller volume were not associated with a greater risk of second BC compared to CHT only (HR = 0.87; 95% CI 0.28–2.66; *p* = 0.80). Patients under the age of 18 were also enrolled.

Two systematic reviews were published by the Cochrane library [36,41] that focused on the risk of second cancer in cHL patients and also investigated the role of different volumes and doses of RT on the incidence of second tumors. Franklin et al. [41] showed no differences in second tumor incidence between EFRT and IFRT. The same conclusions were reached in an updated analysis in 2017 (Peto OR 0.86, 95% CI, 0.64 to 1.16, low quality of evidence), but with a bias of a too short follow-up of the included studies to record the occurrence of solid tumors [36].

According to the Newcastle–Ottawa scale (NOS), the quality of the five retrospective studies was in general low because, although the representativeness and selection of the cHL patients and the duration of the follow-up were good, as was the assessment of the impact of the different radiation volume on the outcome (incidence of second cancers), in particular for the De Bruin [39] and Conway [40] papers, patients under the age of 18 years were also enrolled. According to AMSTAR guidelines, the quality of the review was high for the items considered. 

### 3.3. Are Planned Follow-Up/Screening Schemes Effective in the Management and Early Diagnosis of Second Cancers in cHL or DLBCL Long-Term Survivors Treated, Regardless of the Type of CHT/RT (First and Second Line including ASCT)?

**Figure 3:**
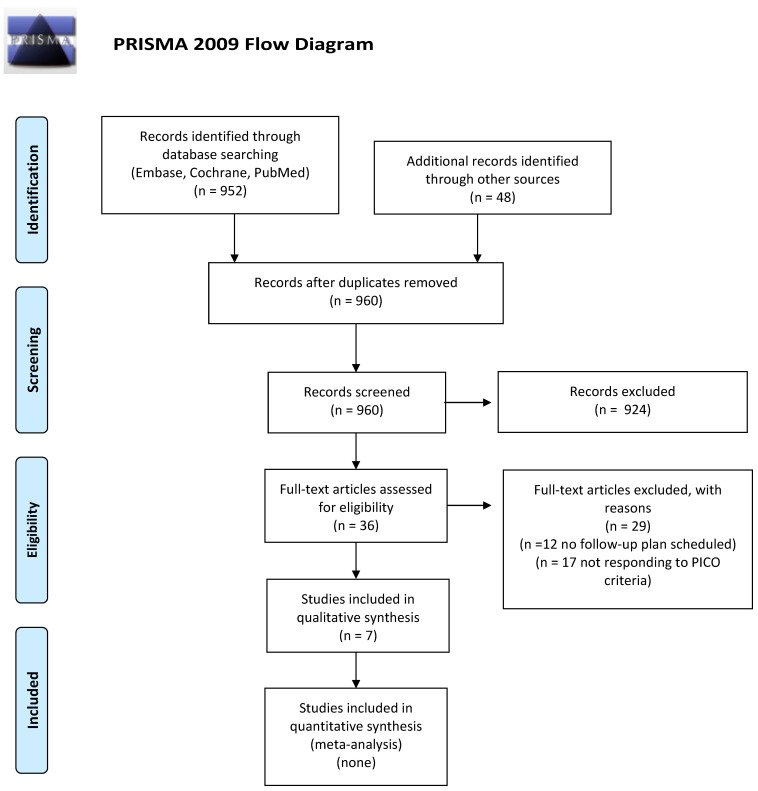
PICO C: PRISMA flow-chart for planned follow-up/screening schemes for the management and early diagnosis of second cancer.

We screened 252 abstracts, and then 22 relevant publications were retrieved as full text. Of these, 15 were excluded and seven studies were included in the final sample and relative analysis. Of the seven included studies, there were no RCTs, five were retrospective, and two systematic reviews. Details of the screening process, including reasons for full-text exclusion, are reported in Figure 3 (PRISMA flow-chart). The results are summarized in Table 2.

Of the 36 papers eligible for full-text examination, only three involved NHL, and two concerned DLBCL without description of detailed planned follow-up for SM beyond 5 years. For this reason, they did not meet the criteria defined by the present PICO. All seven manuscripts selected and included for the qualitative analysis had been conducted in cHL survivors, previously treated with supradiaphragmatic/chest irradiation +/− CHT, with the aim of assessing the screening program and/or evaluating early detection of secondary BC. These are shown below, ordered on the basis of publication year. 

Diller et al. [42] describe a prospective cohort of 90 female cHL survivors (median age 38 years, range 24–51 years) treated with mantle irradiation as part of treatment more than 8 years before enrollment in the study; only patients with a diagnosis of cHL before age 30 were included (range 13–30 years, median 20 years at diagnosis of cHL). The aims were (i) acquisition of data about patient awareness of BC risk and source of risk information, (ii) collection of data about their screening behavior, and (iii) utility of mammographic screening. A baseline questionnaire revealed that 40% of females were unaware of their increased risk of BC after RT. Those who received BC risk information by an oncologist were more conscious of being at high risk, but only 47% had had a mammography (Mx) in the previous 2 years, mostly patients aged ≥35 years. Participants moreover received written recommendations for breast examination/Mx, and an annual follow-up was conducted. During the study (started in 1995), 10 women experienced 12 BCs (2 BC at beginning and 10 during follow-up, median time 3.1 years; 8 mono and 2 bilateral cancers), with a median age of 43 years (range 29–50 years). The median time from cHL to BC was 19.5 years (range 12–28 years). All BC were ductal (2 in situ, 10 invasive) and evident on Mx; 8/10 invasive BC were node negative. The authors concluded that screening with Mx could detect small, node-negative BC in these patients at high risk. A multivariate analysis revealed that older patients who understood that they were at high risk and received risk information from an oncologist were seven-times more likely to have undergone a Mx in the previous 2 years. In the entire cohort, nine SM different from BC were detected during the follow-up period including thyroid cancer, LC, and sarcoma (two of each), pancreas, stomach, and NHL (one of each); at the time of enrollment three patients already had SM (osteosarcoma, melanoma, and NHL, one of each). The study was in general considered of good quality, with the main bias possibly being represented by the short follow-up (median 3.1 years).

Kwong et al. [43] performed a subsequent evaluation after Bloom et al. [55], where 291 women, treated with thoracic lymph node region irradiation +/− CHT for cHL before the age of 35 years at Stanford were contacted by mail offering information about the late effects of cHL treatment. The 167 women who agreed to participate received information on risks potentially associated with their prior cHL therapy (BC, other SM, heart disease, infections). They were advised to initiate or maintain Mx screening and then randomized to receive early or late telephone counseling. At enrollment the average age was 40.4 years (range 26–55 years) with an average of 16.9 years (range 4.5–32.5 years) after cHL treatment. A total 68.9% (*n* = 115) of the cohort reported a new Mx during the study period, and the available Mx (*n* = 99) were reviewed by two radiologists. While, 60.6% showed high density breast tissue on Mx. The authors observed that breast density decreased with increasing patient age at screening; 17.2% were recalled for further imaging or work-up (higher than the recall rate of 10% reported in the general population screening) and of these, 41% (*n* = 7) underwent biopsies, after additional imaging, with identification of one ductal carcinoma in situ (DCIS) and benign findings in the others. Among the unavailable Mx (*n* = 16/115), three DCIS were diagnosed. In total, four (3.5%) new BC were detected (with microcalcification without palpable abnormality in three cases) in the population under study, from 5 to 23 years after cHL treatment (between 25 and 40 years of age), confirming that this population is at higher risk of BC at an unusually young age and that early Mx screening facilitates early diagnosis. This study was considered at low risk of bias for randomization, attrition, and reporting biases; due to its nature, the risk was unclear for selection, intermediate for performance, and high for detection biases.

Lee et al. [44] published the results of a prospective surveillance study of women treated with supradiaphragmatic RT before the age of 30 years for cHL, to assess a potentially optimal BC screening strategy and to evaluate the characteristics of secondary BC. A total of 360 eligible women were identified (years of treatment for cHL 1968–1997) and invited to participate in the surveillance program, from 1997 to 2006, consisting in monthly self-breast examination and annual clinical follow-up plus Mx, starting 8 years after treatment; 115 females agreed to be seen for at least one visit in a high-risk clinic. While, 91% received EFRT (mantle) and 100 participated in annual surveillance (most had Mx alone), with a median interval of 13 years between treatment and first Mx; breast density was evaluated (as per BI-RADS guidelines) on a four-point scale. Women with an extremely or moderately dense breast (52%) received a combined screening with Mx and magnetic resonance imaging (MRI) to clarify suspicious lesions. Twelve BC were diagnosed after a median of 5 years of surveillance (range 1–9 years); median age at BC diagnosis was 40 years (range 35–41 years), and median latency from lymphoma treatment was 16 years (range 13–28 years). Seven BC presented with palpable findings (6 invasive, 1 DCIS) and five were detected by Mx (1 invasive, 4 DCIS). The RR of BC in the screened cohort was 18.5 compared with the expected incidence of BC in an age-matched general population in Ontario. The authors concluded that a more intensive screening program should be adopted, because, despite earlier initiation of Mx screening, a portion of BC were diagnosed clinically with unfavorable characteristics; hypothesizing MRI for early detection of invasive carcinoma as an adjunct to Mx in this high-risk population. The study was considered of good quality for risk of bias according to the NOS, although there was no description for cases lost to follow-up.

Howell et al. [45] in 2009 issued a report of a United Kingdom screening program instituted in 2003 and addressed to women treated with supradiaphragmatic RT for cHL before 36 years of age, the so-called Notification Risk Assessment and Screening Programme (NRASP). In female survivors more than 8 years after supradiaphragmatic RT for cHL and at least 25 years old, whichever occurred later, the screening protocol proposed: no imaging if less than 25 years; annual MRI ± ultrasound (US) if 25–29 years; Mx ± MRI/US if 30–50 years; and Mx every 3 years if more than 50 years, as for the general population. Potentially eligible subjects were identified through the cancer registry, hospital databases, and follow-up clinics and were invited to participate by letter. Among the 417 women eligible for inclusion in the program, 23 (5.5%) with at least one diagnosis of BC were identified (SIR 2.9 vs. age-matched general population); of the 417 women invited for clinical review, 243 attended the NRASP. Five invasive BC, diagnosed within the NRASP, were node negative, while 7/13 invasive BC identified outside the NRASP (*p* < 0.10) showed the involvement of axillary lymph nodes. The mean latency for BC cases was 19.5 ± 8.35 years. Results suggest that the screening strategy is appropriate for early BC detection, although the number of cases was small, with acceptable biopsy rates. The study was considered of good quality for selection of cohorts and comparability according to the NOS, but the assessment of follow-up was not optimal.

Despite not being totally matched to the current PICO, a paper by Elkin et al. [46] was included in our selection, due to their relevant conclusions about the utility of planned screening. The principal aim of this retrospective matched cohort study was to compare the characteristics and outcomes of BC in women with and without a history of RT for cHL. First, cases of BC diagnosed from 1980 to 2006 after RT for cHL were identified from eight North American hospitals, then matched three-to-one with sporadic BC by age, race, and year of BC diagnosis. Secondary BC cases identified numbered 253, matched with 741 women who had BC and no history of cHL, with a complete matching in 94%. For lymphoma survivors, the median age at cHL diagnosis was 23 years; both cohorts had a median age of 42 years at BC diagnosis (vs. 61 years in general population), with a median latency of 18 years (range 1–42 years) from cHL to BC diagnosis. More than half were treated with extended modality RT before 1980, when RT alone was the main approach in cHL treatment. OS was poorer in cHL survivors vs. subjects with sporadic BC, with a median follow-up of 4.6 vs. 5.2 years, respectively. BC-related mortality did not differ significantly between the two groups, but BC treatment options in cHL survivors were limited by prior exposure to chest irradiation and in some cases also to systemic anthracycline-based CHT. Compared with sporadic BC cases, cHL survivors with secondary BC were more likely to detect BC through Mx screening (40% vs. 33%), at an early stage (61% vs. 42%), and be node negative (39% vs. 25%), but also more likely to present with bilateral disease (6% vs. 2%); synchronous or metachronous. The authors concluded that these relevant findings confirmed the efficacy and utility of planned assessment, supporting close control for contralateral BC in this high-risk population. This case-control study was considered of good quality for the evaluation of RoB, according to the NOS. 

An Italian single-center study published in 2013 by Mariscotti et al. [47] reported the experience of a radiology department that had adopted a surveillance protocol for early follow-up breast imaging of patients who underwent RT for cHL between 1980 and 2010. A total of 54 women were retrospectively analyzed, with the aim of evaluating the RR of developing RT-related BC and studying the features of the diagnosed BC on conventional imaging. The group under evaluation had a mean age at cHL diagnosis of 26.1 years (range 8–71 years), a mean interval between RT and first follow-up imaging of 5.5 years (range 1–18 years), and a mean duration of breast surveillance of 10.8 years (range 2–25 years). The relevance of this experience for our PICO concerns the pre-planned age-based follow-up addressed to BC surveillance, consisting of (i) clinical examination and US (Mx if necessary) for women ≤30 years of age (from September 2009, also with MRI, as suggested); (ii) clinical and US examination plus bilateral Mx if >30 years of age. Through this planned screening, seven invasive BC were diagnosed, of which five were monolateral and two bilateral metachronous (after 14 and 25 years from RT), with a mean latency from RT of 15.1 years (range 3–26 years) and a mean age at BC diagnosis of 42.4 years (range 28–55 years). Six cases were classified as stage T1 and one as stage T4, with two node positive. In conclusion, women treated with RT had a higher risk (RR = 6.2) of developing BC vs. the general population. Imaging showed a high breast density in RT-treated subjects, suggesting the use of MRI under 30 years of age and as a complementary method to US/Mx at over 30 years of age, similarly to women with BRCA mutations. The study was considered of intermediate quality, with an appropriate case definition, while the selection of controls and comparability was not optimal. The main bias could be represented by the lack of description for those lost to follow-up.

Ng et al. [48] performed a prospective evaluation, with the main aim of comparing sensitivity and specificity of annual breast MRI vs. Mx (over a 3-year period) in women treated with RT for cHL, under 35 years of age and more than 8 years after treatment. Between 2005 and 2010, 148 women were enrolled and 134 had at least one screen-set with breast MRI and Mx close together, 111 at least two, and 100 subjects completed screening by the third year. A relevant number of biopsies were performed (63 biopsies/45 women) and 29% (18 biopsies/18 women) were positive for malignancy: eight invasive, nine DCIS, and one phyllodes (MRI alone detected 5 BC, Mx alone 6, both modalities 7). All but one BC were preinvasive or subcentimetric node-negative invasive cancers. No interval BC was detected, and 88% of BC diagnosed were ER-positive, hypothesizing a role for hormonal chemoprevention. In RT-treated cHL survivors, contrarily to women screened for genetic/family risk, MRI was not more sensitive than Mx. The results showed that sensitivity was 68% for Mx, 67% for MRI, and 94% with combined modality. The addition of breast MRI to Mx allowed the detection of five additional early BC missed by Mx, improving the overall sensitivity of detection, with better prospects of intervention. Specificity was 93% for Mx, 94% for MRI, and 90% with combined imaging. The finding of early BC each year during the study points out the importance of systematic BC screening in high-risk cohorts like this one. The study was in general considered of good quality. The evaluation for the selection of the non-exposed cohort and comparability was not applicable.

## 4. Discussion

In recent years, the introduction of new drugs and technical improvements to RT has led to a progressive increase in the OS of many histological subtypes of lymphoma, particularly B-cell NHLs and cHL. These improvements have led to the need for a better understanding of the issues involved in survivorship. This includes knowledge of the factors leading to late toxicities, and the introduction of lifestyles that could possibly prevent or reduce the incidence of such complications and, therefore, ameliorate the quality of life of long surviving patients. 

SM have historically been one of the major causes of morbidity and death in lymphoma survivor patients. The aim of this systematic review was to evaluate the factors leading to a higher risk of SM, particularly for CHT and RT techniques, and to evaluate the impact of early detection policies for SM.

Most of the selected papers for PICO A and all of the papers selected for PICO B and C were regarding cHL survivors. Most papers on NHL were discarded, due to the impossibility of discriminating the histologic type of the population. The reason for the lack of literature regarding the incidence of SM in DLBCL and in NHL in general is not clear. The younger age of most patients and the greater importance of RT as part of the treatment, including awareness of its carcinogenetic potential, may be inferred as the causes of the greater interest shown towards SM in cHL.

The use of CHT as part of the treatment for NHL and cHL has been associated with the incidence of SM, particularly with the administration of alkylating agents. These are part of the vast majority of the combination CHT schedules, of which R-CHOP is presently the gold standard for the treatment of DLBCL [1]. In previous decades, more complex, third generation CHT regimens (e.g., MACOP-B) or dose-dense/dose-intense regimens were introduced, in order to improve the efficacy of CHOP. However, the results of large RCTs did not show any improvement in terms of OS [8], even in the rituximab era. In the selected paper by Frontzek et al. [28], younger, high-risk DLCBL patients showed a similar 10-year PFS, and, interestingly, despite the striking difference in CHT dose intensity, the rate of SM was similar in the standard (9%) and experimental arms (8%). The risk of SM also increased after high-dose CHT followed by ASCT; however, despite the more intensive treatment, the incidence of SM was not significantly higher. In their analysis of 154 patients who received ASCT, Minn et al. [34] found a 15-year cumulative incidence of SM of 12%, but the RR of SM was similar to cHL patients who did not receive ASCT. Age >55 years at diagnosis of cHL was identified by Pingali et al. [32] as a risk factor for SM. 

Mechloretamine, a nitrogen mustard, was the first available antiblastic drug, and its efficacy was investigated both as a single agent and in combination with vincristine, procarbazine, and prednisone (MOPP) as the first combination CHT in cHL. ABVD was developed a decade later [56], and appeared to be less toxic than MOPP in terms of SM [57]. The higher incidence of SM after MOPP was also paired with a higher incidence of sAML: in 1659 patients described by Brusamolino et al. [58], the 15-year actuarial risk of sAML was 2.2% after MOPP, and 10.2% after CMT (MOPP plus RT), while no sAML were observed after ABVD. During the following years, many authors tried to reduce the toxicity of MOPP with alternative schedules and hybrid regimens, such as MOPP/AVD. The results of RCTs established ABVD as a gold standard, which allowed obtaining disease control with a lower risk of SM [9,59]. In the Cochrane analysis published by Franklin et al. [36], the rate of sAML and, for younger patients, SM was lower in patients treated with ABVD or ABVD-like therapies than in patients treated with intensified CHT protocols. This finding may be explained by the presence of alkylating agents in the BEACOPP regimen, namely procarbazine and cyclophosphamide. The correct positioning of BEACOPP as a first-line therapy for cHL is still debated, but many trials have investigated the possible role of a PET-driven approach for deescalation [60] or an intensification approach [61], in order to reduce the higher risks of sAML and SM associated with this treatment. The actual impact of the CHT modification should be investigated. In a large retrospective cohort trial by Schaapveld et al. [27], the risk of SM was not inferior in the patients treated in the period 1989–2000 compared with patients treated before 1989; these results should be weighted with the continuing frequent use of EFRT and higher doses in RT until 2005. The authors reported a 40-year cumulative incidence of SM of 48.5%. As expected, almost all patients received EFRT (mantle RT with/without infradiaphragmatic RT) until 1988, while smaller radiation volumes were adopted in the period between 1989 to 2000. The field reduction (supradiaphragmatic field not including the axilla) was associated with a reduction of the risk of BC, without impact on LC risk. Other authors have also reported a reduction of SM incidence or death for SM in cHL patients treated in more recent times, when IFRT instead of EFRT was introduced in clinical practice, although no specific correlations with dosimetric parameters were analyzed [37,38]. A significant result regarding the decrease of SM incidence (namely BC), when specifically comparing EFRT to IFRT (or more recently introduced INRT/ISRT), was reported in two other papers [39,40]. Therefore, although systematic reviews that focus on the risk of SM in cHL patients were not conclusive [36,41], these clinical data seem reassuring, as regards the decrease of SM incidence due to a reduction of radiation volume, such as IFRT. Furthermore, a radiobiological model for estimating the decrease in risks of BC and LC related with 35 Gy mantle RT compared to 35 Gy IFRT showed a transition from 63% to 21% of reductions in the median excess RR of BC and LC, attributable to radiation [62]. The same results were confirmed by a similar study adopting a dosimetric risk-modeling approach [63]. IFRT is therefore predicted to substantially reduce the risk of secondary BC and LC compared with mantle RT. 

Currently, lymphoma patients are treated with ISRT/INRT, according to ILROG guidelines [10,11], but no robust clinical data are yet available about the eventual impact of this further radiation volume reduction on the incidence of SM. Several studies, using dosimetric parameters as surrogate endpoints for the risk of this late toxicity, have shown that the introduction of smaller RT field sizes, such as INRT/ISRT, may be related to a further reduction of the incidence of SM. INRT was the most effective way to reduce the number of SM in a dosimetric study by Schneider et al. [64]. The estimated relative lifetime attributable risk of cancer induction for a 20-year-old patient relative to a historical mantle treatment was as follows: 0.61 for IFRT 40 Gy, 0.55 for IFRT 30 Gy, and 0.45 for INRT 30 Gy, showing that the reduction of field size by applying INRT has the potential to reduce BC induction by approximately 30%. Moreover, the mean doses to lungs, breasts, and thyroid and the estimated lifetime risks for LC and BC induction due to ISRT adoption were systematically and significantly lower than those from the IFRT [65,66,67]. With the adoption of INRT or ISRT, we obtained dosimetric improvements that are likely to translate into lower rates of RT-induced late toxicities. Consequently, in the near future we will witness a further reduction of RT-related SM in cHL and NHL long-survival patients.

Another strategy for minimizing radiation exposure to OAR is the employment of highly conformal RT techniques, such as intensity-modulated RT (IMRT) or volumetric modulated arc therapy (VMAT), possibly associated with the implementation of breath-hold techniques. To assess whether the current technological changes used in conjunction with dose/volume reduction could translate into a reduced impact on SM incidence, to date, only dosimetric studies are available. According to the published data, breath-hold techniques have been associated with a reduction of risk of SM [64,68]. Consensus has yet to be reached, however, regarding the true impact of IMRT/VMAT techniques in lowering the risk of SM for lymphoma patients, suggested given the low-dose bath and the fact that large areas of the body are outside the radiation field [69]. Several dosimetric studies have suggested caution, due to a probable higher risk of LC and BC with IMRT/VMAT [64,66,68,70,71], while no substantial risk of SM was recorded by others [72,73,74,75]. Using IMRT/VMAT, a larger amount of other normal tissues, such as breast or lung tissue, may receive very low doses to larger volumes in comparison with three-dimensional conformal radiation therapy. However, radiobiological estimates showed that the risk of SM, in particular for BC, was very limited if the dose to the breast is lower than 5 Gy [76,77]. 

Finally, although not largely available in clinical practice, proton therapy significantly decreases the all dose/volume metrics of the OARs, suggesting that intensity-modulated proton therapy could have a high potential to reduce late toxicities such as SM in long-survival lymphoma patients [71,78,79]. Currently, lymphoma patients are treated with radiation doses and volumes based on the modern concepts delivered with sophisticated RT techniques, and to a lesser extent with proton therapy. In this scenario, after individualizing and optimizing the radiation dose for each patient, we may obtain dosimetric gains that may translate into a clinically significant benefit. 

Data from SM risk awareness studies in supradiaphragmatic RT-treated lymphoma survivors showed these patients’ unawareness of being at increased risk of BC compared to the general population. This risk also increases many years after RT treatment, although with the introduction of modern, limited-field, reduced-dose RT, it can reasonably be hoped that the incidence will decrease. 

The perspective of BC detection, at an early stage and node negative, with a suitable screening strategy (as shown in the reported papers regarding cHL survivors) validates studies aiming to find the best imaging and timing. Considering that the mean age at diagnosis of secondary BC in cHL survivors is around 40 years, with detections also at a younger age, and that the risk is higher if RT is performed at age ≤35 years, a screening program should be started earlier than in the general population. It also has to be taken into account that increased risk of secondary BC generally emerges with a latency of 10–15 years and persists beyond 25 years of follow-up [80,81,82]. It, therefore, seems reasonable to propose starting screening for secondary BC from 25 years old or after 8 years (whichever occurs last) from RT treatment for exposure ≥10 Gy. A possible screening plan could include annual ultrasound ±MRI for younger patients (25–35 years old) and ultrasound ±MRI plus mammography for those >35 years old. This aspect has been also strengthened by several national guidelines, under the aegis of the German Society for Hematology and Oncology [83], the American College of Radiology [84], and the North American recommendations [85]. Despite this, in a review from a population-based cancer registry from Ontario, the SM screening for lymphoma patients resulted as inadequate, despite the established cancer-screening interventions [86]. 

Information about second cancer risk with recall years after dismission from the clinic is effective but not completely accepted. It is likely that an immediate planned surveillance program at the beginning of the follow-up period would obtain greater compliance. Tailored screening should be based on the CHT-RT treatment, together with individual risk factors, such as lifestyle habits or family history. 

Other than screening for BC with largely adopted and evidence-based screening tests in lymphoma survivors, the risk of other SM should be addressed, and an optimal screening tool and timing should be identified, according to the individual risk and available literature [16]. 

As regards the risk of secondary LC, which is associated with poor prognosis, especially in subjects with exposure to alkylating agents/RT and tobacco, low-dose computed tomography (LDCT) has been evaluated as a screening tool [87]. The lack of a well-defined interval for LDCT excluded this paper from our systematic review. On the basis of a cost-effectiveness analysis, it seems reasonable to perform an annual chest LDCT scan on smokers with previous cHL treatment as alkylators/RT [88]. In a subsequent cost-effectiveness study, Wattson et al. developed a model to estimate whether screening with LDCT might allow dehemati of early stage/resectable LC. The authors concluded that screening may be cost-effective for all smokers, but possibly not for non-smokers [89]. 

From our systematic review, a lack of evidence for SM screening programs targeting DLBCL survivors also emerged, outlining an additional unmet clinical need.

With the aim of overcoming the gaps in evidence which emerged from the systematic review, the consensus of the authors (including 4 onco-hematologists, 1 radiotherapist, and 2 methodologists) led to the formulation of the following observations, in agreement with the most recent general oncologic guidelines (NCCN 2021) [90]. As mentioned previously, in order to guide the detection of SM, individual risks, including family history, sex, age, anti-cancer treatment, and behavioral risk factors, should be assessed during the follow-up visits. Related to these factors, a thyroid gland ultrasonography should be advised for patients who have received neck RT. A colorectal cancer screening for patients who have received abdominal RT (≥20 Gy), starting from the age of 30 years or 5 years (whichever occurs last), after therapy and in the case of positive family history should also be scheduled [90]. A dermatoscopic evaluation should be addressed annually to patients treated with RT, focusing on the irradiated skin areas (as well as recommending the use of sunscreen of at least SPF 30) [90]. A Pap-test should be advised according to the general gynecologic guidelines [91]. A periodic blood cell count evaluation for the detection of sMDS/sAML should also be considered, especially after dose-intense CHT or ASCT. 

The authors highlight the need to increase awareness in lymphoma survivors and clinicians about the value of follow-up tests, which could improve the quality and success of care for patients already cured of lymphoma. Follow-up of the patient within dedicated outpatient survivorship programs and the use of tailored survivorship care plans should guarantee more complete attention is given to the patient and increased compliance [2,14]. Moreover, optimal screening programs for SM other than BC should be explored by future research studies. A summary of the suggestions for SM monitoring strategies is given in Table 3.

## 5. Conclusions

In conclusion, SM incidence has been studied more frequently in patients with cHL than in patients with DLBCL. Increasing knowledge of the carcinogenetic effects of alkylating agents has led to the progressive dismissal of older CHT regimens, leaving ABVD and BEACOPP as the most used first-line CHT regimens for cHL. R-CHOP is currently the gold standard for the treatment of DLCBL. The impact of novel therapies, such as checkpoint inhibitors, brentuximab vedotin, and CAR-T still needs to be assessed.

Moreover, lymphoma patients are currently treated with radiation doses and volumes based on modern concepts, and RT is delivered with sophisticated techniques, including proton therapy in a small number of patients. In this scenario, after individualizing and optimizing the radiation dose/volume for each patient, we can obtain dosimetric gains that may translate into a clinically significant benefit, also in terms of a reduction of SM incidence.

Future efforts of the FIL ‘Long-term Survivor Committee’ researchers will be focused on applying screening evidence and the recommendations that emerged from this systematic review to a tertiary prevention strategy aimed at lymphoma survivors. The aim is to carry out an early diagnosis of SM and reduce their incidence by removing/reducing known and modifiable risk factors in the cHL and DLBCL long survival setting.

## Figures and Tables

**Table 1:** Clinical questions and corresponding PICOs addressed by this systematic review.

Clinical Question	PICOs
PICO A: What is the incidence of SM in cHL or DLBCL long-term survivors after first or second line treatments?	P: population of cHL or DLBCL long-term survivors (≥5 years of disease/treatment free) aged ≥ 18 years at diagnosis I: chemotherapy (e.g., ABVD for cHL; R-CHOP for DLBCL), second-line chemotherapy and autologous transplant, radiotherapy C1: none C2: general population equal to age and sex C3: other chemotherapy/radiotherapy treatment regimens O: number of cases of second neoplasms (breast, lung, gastrointestinal, prostate, melanoma, thyroid, etc.)
PICO B: Has the incidence of SM in cHL or DLBCL long-term survivors who underwent first or second line chemotherapy and ASCT changed with the introduction of modern radiotherapy?	P: population of cHL or DLBCL long-term survivors (≥5 years of disease/treatment free) aged ≥ 18 years at diagnosis I: new radiotherapy approaches (e.g., 3DCRT, volume reduction, dose reduction, IMRT) C: previous radiotherapy regimens (EFRT, 2DRT, RT dose) O: number of cases of second malignancies (e.g., breast, lung, gastrointestinal, prostate, melanoma, thyroid)
PICO C: Are planned follow-up/screening schemes effective for the management and early diagnosis of second cancers in cHL or DLBCL long-term survivors treated, regardless of the type of CHT/RT (first and second line including ASCT)?	P: population of cHL or DLBCL long-term survivors (≥5 years of disease/treatment free) aged ≥18 years at diagnosis I: scheduled follow-up/monitoring schemes for second malignancies, diversified by age, gender/sex, which include laboratory and instrumental tests (e.g., breast mammography/MRI, chest CT, stool occult blood, colonoscopy, dermatoscopy, thyroid US, PSA) C1: no follow up/monitoring planned C2: follow up/monitoring schemes with different intensity (with respect to I, including different times and frequencies) O: early diagnosis of second neoplasms, problems related to overdiagnosis, QoL, mortality

ASCT, autologous stem cell transplant; cHL, classical Hodgkin lymphoma; DLBCL, diffuse large B-cell lymphoma; EFRT, extended field radiation therapy; IMRT, intensity modulated radiation therapy; MRI, magnetic resonance imaging; PICO: P, population; I, intervention; C, control; O, outcome; PSA, prostate specific antigen; RT, radiation therapy; QoL, quality of life; SM, second malignancies; US, ultrasound; 2DRT, 2-dimension radiation therapy; 3DCRT, 3-dimensional conformal radiation therapy.

**Table 2:** Summary of results.

Study	Study Design and Sample Size	Intervention and Comparison	Main Outcomes
PICO 1			
André M, 2020 [24]	Pooled analysis of randomized control trials (1227 patients with advanced stage cHL in first line	BEACOPP vs. ABVD	Incidence of SM per 1000 person-years was 6.3% in ABVD arm vs. 9.6% in BEACOPP arm. Thirteen cases of secondary MDS/AML were reported in the BEACOPP arm, compared to none in the ABVD arm.
Torok JA, 2015 [25]	Retrospective cohort study (90 patients with early unfavourable cHL in first line)	CT (ABVD in 88%) plus RT	Seven SM were diagnosed in the cohort. The median time for diagnosis of SM was 10 years (range: 1–14 years), but all hematological SM occurred within 3 years of diagnosis.
Engert A, 2007 [26]	Randomized control trial (627 patients with early favourable cHL in first line)	2 ABVD plus EFRT 30 Gy vs. EFRT 30 Gy plus boost 10 Gy	Total number of SM was 39 (6.2%). Eleven of the solid SM occurred in irradiated areas, three in nonirradiated areas, and for seven cases it was unknown or unclear. There were no significant differences between treatment arms. Between years 2 and 9, the incidence remained constant (0.8% per year); numbers at risk were too small for reliable estimates beyond year 9.
Schaapveld M, 2015 [27]	Retrospective cohort study (3905 patients with cHL in first line)	CT and/or RT	A total 1055 SM were observed in 908 patients; a third cancer developed in 130 patients, and a fourth developed in 17. SM risk in cHL treated patients was higher than in the general population (121.8 excess cancers per 10,000 PY). Breast cancer contributed most (24.9 cases per 10,000 PY in the whole cohort, 54.3 cases per 10,000 PY in women. SIR for any SM remained high for at least 35 years after the start of treatment for cHL, whereas the absolute excess risk increased steadily over time. A cumulative procarbazine dose of 4.3 g or more per square meter of body-surface area was associated with a higher risk of gastrointestinal cancer.
Frontzek F, 2021 [28]	Randomize control trial (275 patients with high-risk DLBCL in first line)	R-megaCHOEP vs. R-CHOEP14	Twenty-two SM were reported in the ITT population, 9% in the R-CHOEP14 group and 8% in the R-MegaCHOEP group. The median time to MDS/AML was 44 months; median time to solid SM was 72 months. Age older than 50 years at diagnosis was the only factor associated with significantly increased risk of secondary SM.
Coiffier B, 2010 [29]	Randomized control trial (399 patients with DLBCL in first line, 60–80 years old)	R-CHOP21 vs. CHOP21	Fourty-three patients (10.8%) developed a SM after entering the study, 22 in the CHOP arm and 21 in the R-CHOP arm. Three patients developed a third SM, 1 in the CHOP arm and 2 in the R-CHOP arm. There was no pattern in the type of secondary cancers that occurred.
Castellino A, 2018 [30]	Pooled analysis of prospective trial (112 patients with DLBCL in first line)	R-CHOP21 plus lenalidomide	SM were observed in seven patients (6.3%). The median time from the end of treatment to the SM onset was 16.4 months.
Paudel N, 2019 [31]	Retrospective cohort study (89 patients with relapsed/refractory cHL)	Total lymphoid irradiation plus carboplatin-cyclophosphamide- etoposide and ASCT	Eight of the 89 patients had developed SM at a median of 5.6 years from the time of transplant. Five patients developed hematologic SM, three were solid SM. Three of the patients with subsequent hematologic SM had received MOPP.
Pingali SR, 2017 [32]	Retrospective cohort study (310 patients with relapsed/refractory cHL patients)	ASCT with BEAM conditioning, mobilization with ifosfamide and etoposide	Cumulative incidence of SM was 11% in the entire cohort, with 13 cases of MDS/AML, 5 non-melanoma skin cancers, 1 prostate cancer, 1 MPN, 1 sarcoma. The incidence of SM was significantly higher in pts aged >55 years at diagnosis, even after exclusion of second skin cancers. Time from initial diagnosis to transplantation and exposure to RT were not significant predictors of SM in the study population. MOPP, which was frontline CT in 20 patients, all of whom were <55 years of age, was associated with higher incidence of SM compared with ABVD.
Sibon D, 2016 [33]	Retrospective cohort study (245 patients with relapsed/refractory cHL)	Poor risk: tandem ASCT; other patients single ASCT with BEAM conditioning	Sixteen SM occurred. The 10- and 15-year cumulative incidences of SM were 8% and 15%, respectively; the 10-year cumulative incidences of SM were 15% in intermediate-risk patients and 1.5% in poor-risk patients. Considering only patients who did not relapse after completing ASCT, the 10- and 15-year cumulative incidences of SM were 9% and 13%, respectively; the 10- and 15-year cumulative incidences of SM were 16% and 24%, respectively, for the intermediate-risk patients and 2% and 2% for the poor-risk patients.
Minn AY, 2012 [34]	Retrospective cohort study (154 patients with relapsed/refractory cHL)	Etoposide + cyclophosmamide with TBI or carmustin or lomustin	There were 20 SM, 18 occurring more than 2 years from ASCT. The 5-, 10-, and 15-year cumulative incidence of SM was 5%, 8%, and 12%, respectively. AER was 160 per 10,000 PY of follow-up. The risk of SM compared with patients with cHL in the SEER registry was not elevated 5–10 years after ASCT, but was higher 2–5 and >10 years after ASCT. The overall risk of SM was 3.0 compared with cHL patients from SEER with an AER of 123 per 10,000 PY of follow-up. Overall risk of SM among patients receiving ASCT was not elevated compared with the non-ASCT population at Stanford.
Tarella C, 2011 [35]	Retrospective cohort study (234 patients with relapsed/refractory cHL; 569 patients with high-risk DLBCL in first line or relapsed/refractory DLBCL)	High-dose sequential chemotherapy	The cumulative incidence of MDS/AML in cHL at 5-, 10, and 15-years was 4.1%, 5%, and 15%, respectively, while the cumulative incidence of solid SM was 1.8, 5.5, and 6.9%, respectively. Risk analyses for MDS/AML and solid SM were performed for the whole populations and not for cHL patients only. The overall SIR for MDS/AML was 2.6, with a significantly higher risk for patients aged younger than 45 years (SIR 7.2) and 45 to 65 years of age (SIR 2.1) compared to the age-matched Italian population. Overall, the SIR for solid SM was 3.2; again, increased risk compared to the Italian population was documented for patients aged younger than 45 years (SIR 7.6) and 45 to 65 years of age (SIR 2.7).
Franklin J, 2017 [36]	Systematic review (9498 patients with cHL in first line)	Different therapies including CT and RT	In patients treated with CT the omission of additional RT probably reduces SM incidence, corresponding to an estimated reduction of 8-year SM risk from 8% to 4%. The authors observed that the decrease would be particularly true for AML. The authors investigated the role of the number of CT courses, which probably has little or no effect on SM risk, and the role of intensified CT in patients with advanced stage disease, with insufficient evidence to determine the effect on SM. In patients who received intensified CT, the rate of secondary AML (and for younger patients, all SM) was probably higher than in patients treated with standard-dose ABVD-like protocols.
PICO 2			
Patel CG, 2018 [37]	Retrospective cohort study (1541 patients with early stage cHL in first line)	IFRT or EFRT	A trend of lower risk of death from SM in more recently-treated patients (smaller fields) was observed, though this did not reach statistical significance, likely due to the small number of events within each treatment era.
LeMieux MH, 2015 [38]	Retrospective cohort study (8807 patients with early stage cHL in first line)	IFRT or EFRT	The authors suggests that patients treated with RT prior to 2000 (larger fields) had a slightly higher risk of SM compared to treatment in 2000 and later.
Schaapveld M, 2015 [27]	Retrospective cohort study (3905 patients with cHL in first line)	CT and/or RT	Patients who received supradiaphragmatic RT not including the axilla had a lower risk of a solid SM than those who received complete mantle-field RT, largely due to a lower risk of breast cancer.
De Bruin ML, 2009 [39]	Retrospective cohort study (1022 female patients with cHL in first line)	Mantle field RT or smaller field RT	Mantle field RT (involving the axillary, mediastinal, and neck nodes) was associated with a 2.7-fold increased risk compared with similarly dosed (36 to 44 Gy) mediastinal RT alone.
Conway JL, 2017 [40]	Retrospective cohort study (734 patients with cHL in first line)	Mantle field RT or smaller field RT	The 20-year cumulative incidences for secondary breast cancer, after accounting for death and loss to follow-up as competing risks, were 7.5% in mantle field and 3.1% in smaller field RT, compared to 2% in CT only.
Franklin J, 2017 [36]	Systematic review (9498 patients with cHL in first line)	Different therapies including CT and RT	There is insufficient evidence to determine whether smaller radiation field reduces SM risk and the impact on OS and PFS, with the bias of a too short follow-up of the included studies to record the occurrence of solid tumors.
Franklin J, 2005 [41]	Systematic review (9312 patients with cHL in first line)	Different therapies including CT and RT	No difference in second tumor incidence between EFRT and IFRT.
PICO 3			
Diller L, 2002 [42]	Prospective cohort study (90 partecipating out of 167 patients with cHL treated with RT)	Mx	Ten out of 90 women had a breast cancer, 8 unilateral and 2 bilateral cancer. All cancers were detectable with Mx. Most of the tumors were small and without evidence of nodal involvement. Nine non-breast cancers have developed in members of this cohort during the follow-up period. Screening by Mx can detect small, node-negative breast cancers in these patients. Multivariate analysis revealed that older patients who understood that they were at high risk and received risk information from an oncologist were seven times more likely than patients without this profile to have had a mammogram in the previous 2 years
Kwong A, 2008 [43]	Randomized trial (167 responding out of 297 patients with cHL treated with RT)	Early vs. late telephone counseling	Completion of Mx during the period of the study was reported by 115 of the 167 subjects: 99 Mx were reviewed, 17 recalled; 7 of the 17 women with abnormal Mx were recommended for biopsy. Four out of 115 (3.5%) women who reported completion of Mx (3.5%) were diagnosed with DCIS and two of these had, at least, microscopic evidence for invasive cancer. Three of these patients were diagnosed solely because of calcifications observed on mammography. In the fourth, mammography confirmed suspicious findings noted on self-examination and professional clinical examination.
Lee L, 2008 [44]	Prospective cohort study (100 responding out of 360 patients with cHL treated with RT)	Clinical exam and Mx	Twelve of the 100 participating women (12%) were diagnosed with breast cancer after a median of 5 years of surveillance. Seven cancers presented as palpable masses (six invasive, one DCIS), five were detected by Mx (one invasive, four DCIS). Screening Mx may be effective at detecting DCIS, but may be inadequate for early invasive BC detection in this high-risk population. Evaluation of more intensive screening and the contribution of MRI for earlier detection is warranted.
Howell SJ, 2009 [45]	Report of screening (243 screening reports in patients with cHL treated with RT)	No imaging or breast MRI/US or Mx/US or Mx	Of 417 women, 23 (5.5%) have been diagnosed with breast cancer. Five of them were diagnosed within the screening program, none of them involved axillary lymph nodes compared with 7 of 13 (54%) diagnosed outside the program.
Elkin EB, 2011 [46]	Observational case-control study (253 patients with cHL treated with RT matched 741 breast cancer patients with no history of cHL)	Various surveillance methods	cHL survivors were more likely to have breast cancer detected by screening Mx (40% vs. 33%), were more likely to be diagnosed at an earlier stage (61% vs. 42%), were less likely to have axillary lymphnode involvement (25% vs. 39%), and were more likely to present with bilateral disease (6% vs. 2%).
Mariscotti G, 2013 [47]	Retrospective cohort trial (54 patients with cHL treated with RT)	Clinical exam/US or clinical exam/US/Mx	The authors concluded that patients treated with RT have a higher risk of developing breast cancer and that they need to undergo adequate breast surveillance protocols. They suggested to use of MRI in patients ≤30 years of age and as a complementary method to US/Mx in patients >30, as suggested for women with BRCA mutations.
Ng AK, 2013 [48]	Prospective cohort study (148 patients with cHL treated with RT)	MRI and Mx surveillance	The overall sensitivity of Mx for breast cancer detection was 68%, as compared with 67% for breast MRI. The use of both screening modalities increased the sensitivity to 94%

AER, absolute excess risk; ASCT, autologous stem cell transplant; cHL, classical Hodgkin lymphoma; CT, chemotherapy; DCIS, ductal carcinoma in situ; DLBCL, Diffuse large B-cell lymphoma; EFRT, extended field radiation therapy; ITT, intention to treat; MDS/AML, myelodysplastic syndromes/acute myeloid leukemia; MPN, myeloproliferative neoplasms; MRI, magnetic resonance imaging; Mx, mammography; OS, overall survival; PFS, progression-free survival; PY, person-year; RT, radiation therapy; SIR, standardized incidence ratio; SM, second malignancies; US, ultrasound.

**Table 3:** Highlights emerging from the systematic review and expert panel advice: suggested follow-up schemes for early diagnosis of second malignancies.

Monitoring strategies should be individualized, depending on RT dose, type of CT regimen, age at therapy, and predisposing factors (family history, sex, behavioral risk factors).
No evidence of screening programs for DLBCL survivors,
Breast cancer: for patients treated with >10 Gy RT on the chest: start at age 40 or 8 years after RT, whichever comes first, by annual Mx, add annual breast MRI for women who received chest RT between ages 10–30 years.
Lung cancer: annual chest LDCT scan for smokers treated with alkylators/RT.
Skin cancer: annual skin evaluation of the irradiated skin areas.
Thyroid cancer: neck ultra-sound for pts treated with neck RT.
Colorectal cancer: annual FOB and colonoscopy every 10 years (based on findings) for pts treated with abdominal RT (≥20 Gy), starting from the age of 30 years or 5 years after RT.
MDS/AML: annual blood cell count evaluation.

CT, chemotherapy; DLBCL, diffuse large B-cell lymphoma; LDCT, low-dose chest computed tomography; MDS/AML, myelodysplastic syndromes/acute myeloid leukemia; MRI, magnetic resonance imaging; Mx, mammography; RT, radiation therapy.

## Supplementary Materials

The following are available online at https://www.mdpi.com/article/10.3390/cancers14030519/s1. Data S1: Search strategies; Table S1: Data selection and extraction; Table S2: Risk of bias.

## References

[1] Sehn L.H., Salles G. (2021). Diffuse large B-cell lymphoma. N. Engl. J. Med..

[2] National Cancer Institute: Surveillance, Epidemiology, and End Results Program. https://seer.cancer.gov/statfacts/html/hodg.html.

[3] Bonadonna G., De Lena M., Banfi A., Lattuada A. (1973). Secondary neoplasms in malignant lymphomas after intensive therapy. N. Engl. J. Med..

[4] Minoia C., Bari A., Nassi L., Banzi R., Gerardi C., Lenti V., Calabrese M., Spina M., Guarini A. (2021). Management of lymphoma survivor patients in Italy: An evaluation by Fondazione Italiana Linfomi. Tumori.

[5] Travis L.B., Rabkin C.S., Brown L.M., Allan J.M., Alter B.P., Ambrosone C.B., Begg C.B., Caporaso N., Chanock S., DeMichele A. (2006). Cancer survivorship–genetic susceptibility and second primary cancers: Research strategies and recommendations. J. Natl. Cancer Inst..

[6] Bessell E.M., Bouliotis G., Armstrong S., Baddeley J., Haynes A.P., O’Connor S., Nichols-Elliott H., Bradley M. (2012). Long-term survival after treatment for Hodgkin’s disease (1973–2002): Improved survival with successive 10-years cohorts. Br. J. Cancer.

[7] Coiffier B., Lepage E., Brière J., Herbrecht R., Tilly H., Bouabdallah R., Morel P., Van Den Neste E., Salles G., Gaulard P. (2002). CHOP chemotherapy plus rituximab compared with CHOP alone in elderly patients with diffuse large B-cell lymphoma. N. Engl. J. Med..

[8] Fisher R.I., Gaynor E.R., Dahlberg S., Oken M.M., Grogan T.M., Mize E.M., Glick J.H., Coltman C.A., Miller T.P. (1993). Comparison of a standard regimen (CHOP) with three intensive chemotherapy regimens for advanced non-Hodgkin’s lymphoma. N. Engl. J. Med..

[9] Duggan D.B., Petroni G.R., Johnson J.L., Glick J.H., Fisher R.I., Connors J.M., Canellos G.P., Peterson B.A. (2003). Randomized comparison of ABVD and MOPP/ABV hybrid for the treatment of advanced Hodgkin’s disease: Report of an Intergroup trial. J. Clin. Oncol..

[10] Specht L., Yahalom J., Illidge T., Berthelsn A.K., Constine L.S., Eich H.T., Girinsky T., Hoppe R.T., Mauch P., Mikhaeel N.G. (2014). ILROG. Modern radiation therapy for Hodgkin lymphoma: Field and dose guidelines from the International Lymphoma Radiation Oncology Group (ILROG). Int. J. Radiat. Oncol. Biol. Phys..

[11] Yahalom J., Illidge T., Specht L., Hoppe R.T., Li Y., Tsang R., Wirth A., International Lymphoma Radiation Oncology Group (2015). Modern radiation therapy for Hodgkin lymphomas: Field and dose guidelines from the International Lymphoma Radiation Oncology Group. Int. J. Radiat. Oncol. Biol. Phys..

[12] Chiappella A., Martelli M., Angelucci E., Brusamolino E., Evangelista A., Carella A.M., Stelitano C., Rossi G., Balzarotti M., Merli F. (2017). Rituximab-dose-dense chemotherapy with or without high-dose chemotherapy plus autologous stem-cell transplantation in high-risk diffuse large B-cell lymhpoma (DLCL04): Final results of a multi-center, open-label, randomized, controlled, phase 3 study. Lancet Oncol..

[13] Van Leeuwen F.E., Ng A.K. (2016). Long-term risk of second malignancies and cardiovascular disease after Hodgkin lymphoma treatment. Hematol. Am. Soc. Educ. Program.

[14] Ciavarella S., Minoia C., Quinto A.M., Oliva S., Carbonara S., Cormio C., Cox M.C., Bravo E., Santoro F., Napolitano M. (2017). Improving care provision for lymphoma long-term survivors. Clin. Lymphoma Myeloma Leuk..

[15] Gerardi C., Allocati E., Minoia C., Guarini A., Banzi R. (2021). Long-term follow-up of classical Hodgkin lymphoma and diffuse large B-cell lymphoma survivors: Aims and methodological approach for Fondazione Italiana Linfomi systematic reviews. Cancers.

[16] Minoia C., Gerardi C., Allocati E., Daniele A., De Sanctis V., Bari A., Guarini A. (2021). The Impact of Healthy Lifestyles on Late Sequelae in Classical Hodgkin Lymphoma and Diffuse Large B-Cell Lymphoma Survivors. A Systematic Review by the Fondazione Italiana Linfomi. Cancers.

[17] Franceschetti S., Annunziata M.A., Agostinelli G., Gerardi C., Allocati E., Minoia C., Guarini A. (2021). Late Neurological and Cognitive Sequelae and Long-Term Monitoring of Classical Hodgkin Lymphoma and Diffuse Large B-Cell Lymphoma Survivors: A Systematic Review by the Fondazione Italiana Linfomi. Cancers.

[18] Viviani S., Caccavari V., Gerardi C., Ramadan S., Allocati E., Minoia C., Guarini A., Di Russo A. (2021). Male and Female Fertility: Prevention and Monitoring Hodgkin’ Lymphoma and Diffuse Large B-Cell Lymphoma Adult Survivors. A Systematic Review by the Fondazione Italiana Linfomi. Cancers.

[19] Minoia C., Gerardi C., Allocati E., De Sanctis V., Franceschetti S., Viviani S., Annunziata M.A., Bari A., Skrypets T., Oliva S. (2021). Late toxicities and long-term monitoring in classical Hodgkin lymphoma and diffuse large B-cell lymphoma survivors: A series of systematic reviews of the Fondazione Italiana Linfomi. Hematol. Oncol..

[20] Moher D., Liberati A., Tetzlaff J., Altman D.G. (2009). The PRISMA group. Preferred reported items for systematic reviews and meta-analysis. The PRISMA statement. PLoS Med..

[21] Shea B.J., Reeves B.C., Wells G., Thuku M., Hamel C., Moran J., Moher D., Tugwell P., Welch V., Kristjansson E. (2017). AMSTAR 2: A critical appraisal tool for systematic reviews that include randomised or non-randomised studies of healthcare interventions, or both. Br. Med. J..

[22] Higgins J.P., Altman D.G., Higgins J.P., Green S. (2010). The Cochrane Collaboration’s tool for assessing risk of bias. Cochrane Handbook for Systematic Reviews of Interventions.

[23] Wells Ga S.B., O’connell D., Peterson J., Welch V., Losos M., Tugwell P. (1999). The Newcastle-Ottawa Scale (NOS) for Assessing the Quality of Non-Randomised Studies in Meta-Analyses.

[24] Andre M.P.E., Carde P., Viviani S., Bellei M., Fortpied C., Hutchings M., Gianni A.M., Brice P., Casasnovas O., Gobbi P.G. (2020). Long-term overall survival and toxicities of ABVD vs BEACOPP in advanced Hodgkin lymphoma: A pooled analysis of four randomized trials. Cancer Med..

[25] Torok J.A., Wu Y., Prosnitz L.R., Kim G.J., Beaven A.W., Diehl L.F., Kelsey C.R. (2015). Low-dose consolidation radiation therapy for early stage unfavourable Hodgkin lymphoma. Int. J. Radiat. Oncol. Biol. Phys..

[26] Engert A., Franklin J., Eich H.T., Brillant C., Sehlen S., Cartoni C., Herrmann R., Pfreundschuh M., Sieber MTesch H., Franke A. (2007). Two cycles of doxorubicin, bleomycin, vinblastine, and dacarbazine plus extended-field radiotherapy is superior to radiotherapy alone in early favorable Hodgkin’s lymphoma: Final results of the GHSG HD7 trial. J. Clin. Oncol..

[27] Schaapveld M., Aleman B.M.P., van Eggermond A.M., Janus C.P.M., Krol A.D.G., van del Maazen R.W.M., Roesink J., Raemaekers J.M.M., de Boer J.P., Zijlstra J.M. (2015). Second cancer risk up to 40 years after treatment for Hodgkin’s lymphoma. N. Engl. J. Med..

[28] Frontzek F., Ziepert M., Nickelsen M., Altmann B., Glass B., Haenel M., Truemper L., Held G., Bentz M., Borchmann P. (2021). Rituximab plus high-dose chemotherapy (MegaCHOEP) or conventional chemotherapy (CHOEP14) in young, high-risk patients with aggressive B-cell lymphoma: 10-year follow-up of a randomized, open-label, phase 3 trial. Lancet Haematol..

[29] Coiffier B., Thieblemont C., Van Den Neste E., Lepeu G., Plantier I., Castaigne S., Lefort S., Marit G., Macro M., Sebban C. (2010). Long-term outcome of patients in the LNH-98.5 trial, the first randomized study comparing rituximab-CHOP to standard CHOP chemotherapy in DLBCL patients: A study by the Groupe d’Etudes des Lymphomes de l’Adulte. Blood.

[30] Castellino A., Chiappella A., LaPlant B.R., Pederson L.D., Gaidano G., Macon W.R., Inghirami G., Reeder C.B., Tucci A., King R.L. (2018). Lenalidomide plus R-CHOP21 in newly diagnosed diffuse large B-cell lymphoma (DLBCL): Long-term follow-up results from a combined analysis from two phase 2 trials. Blood Cancer J..

[31] Paudel N., Schulze D., Gentzler R.D., Evens A.M., Helenowski I., Dillehay G., Frankfurt O., Mehta J., Donnelly E.D., Gordon L.I. (2019). Patterns of failure and survival outcomes after total lymphoid irradiation and high-dose chemotherapy with stem cell transplantation for relapsed or refractory classical Hodgkin lymphoma. Int. J. Radiat. Oncol. Biol. Phys..

[32] Pingali S.R., Saliba R.M., Anderlini P., Hosing C., Khouri I., Alousi A.M., Niego Y., Qazilbash M.H., Champlin R., Popat U.R. (2017). Age over Fifty-Five Years at Diagnosis Increases Risk of Second Malignancies after Autologous Transplantation for Patients with Hodgkin Lymphoma. Biol. Blood Marrow Transplant..

[33] Sibon D., Morschhauser F., Resche-Rigon M., Ghez D., Dupuis J., Marçais A., Deau-Fischer B., Boubdallah R., Sebban C., Salles G. (2016). Single or tandem autologous stem-cell transplantation for first-relapsed or refractory Hodgkin lymphoma: 10-year follow-up of the prospective H96 trial by the LYSA/SFGM-TC study group. Haematologica.

[34] Minn A.Y., Riedel E., Halpern J., Johnston L.J., Horning S.J., Hoppe R.T., Goodman K.A. (2012). Long-term outcomes after high-dose therapy and autologous haematopoietic cell rescue for refractory/relapsed Hodgkin lymphoma. Br. J. Haematol..

[35] Tarella C., Passera R., Magni M., Benedetti F., Rossi A., Gueli A., Patti C., Parvis G., Ciceri F., Gallamini A. (2011). Risk factors for development of secondary malignancy after high-dose chemotherapy and autograft, with or without rituximab: A 20-year retrospective follow-up study in patients with lymphoma. J. Clin. Oncol..

[36] Franklin J., Eichenauer D.A., Becker I., Monsef I., Engert A. (2017). Optimization of chemotherapy and radiotherapy for untreated Hodgkin lymphoma patients with respect to second malignant neoplasms, overall and progression-free survival: Individual participant data-analysis (review). Cochrane Database Syst. Rev..

[37] Patel C.G., Michaelson E., Chen Y.H., Silver B., Marcus K.J., Stevenson M.A., Mauch P., Ng A.K. (2018). Reduced Mortality Risk in the Recent Era in Early-Stage Hodgkin Lymphoma Patients Treated With Radiation Therapy With or Without Chemotherapy. Int. J. Radiat. Oncol. Biol. Phys..

[38] LeMieux M.H., Solanki A.A., Mahmood U., Chmura S.J., Koshy M. (2015). Risk of second malignancies in patients with early-stage classical Hodgkin’s lymphoma treated in a modern era. Cancer Med..

[39] De Bruin M.L., Sparidans J., van’t Veer M.B., Noordijk E.M., Louwman M.W.J., Zijlstra J.M., van den Berg H., Russell N.S., Broeks A., Baaijens M.H.A. (2009). Breast Cancer Risk in Female Survivors of Hodgkin’s Lymphoma: Lower Risk After Smaller Radiation Volumes. J. Clin. Oncol..

[40] Conway J.L., Connors J.M., Tyldesley S., Savage K.J., Campbell B.A., Zheng Y.Y., Hamm J., Pickles T. (2017). Secondary Breast Cancer Risk by Radiation Volume in Women with Hodgkin Lymphoma. Int. J. Radiat. Oncol. Biol. Phys..

[41] Franklin J., Paus M.D., Pluetschow A., Specht L. (2005). Chemotherapy, radiotherapy and combined modality for Hodgkin’s disease, with emphasis on second cancer risk (Review). Cochrane Database Syst. Rev..

[42] Diller L., Medeiros Nancarrow C., Shaffer K., Matulonis U., Mauch P., Neuberg D., Tarbell N.J., Litman H., Garber J. (2002). Breast cancer screening in women previously treated for Hodgkin’s disease: A prospective cohort study. J. Clin. Oncol..

[43] Kwong A., Hancock S.L., Bloom J.R., Pal S., Birdwell R.L., Mariscal C., Ikeda D.M. (2008). Mammographic screening in women at increased risk of breast cancer after treatment of Hodgkin’s disease. Breast. J..

[44] Lee L., Pintilie M., Hodgson D.C., Goss P.E., Crump M. (2008). Screening mammography for young women treated with supradiaphragmatic radiation for Hodgkin’s lymphoma. Ann. Oncol..

[45] Howell S.J., Searle C., Goode V., Gardener T., Linton K., Cowan R.A., Harris M.A., Hopwood P., Swindell R., Norman A. (2009). The UK national breast cancer screening programme for survivors of Hodgkin lymphoma detects breast cancer at an early stage. Br. J. Cancer.

[46] Elkin E.B., Klem M.L., Gonzales A.M., Ishill N.M., Hodgson D., Ng A.K., Marks L.B., Weidhaas J., Freedman G.M., Miller R.C. (2011). Characteristics and outcomes of breast cancer in women with and without a history of radiation for Hodgkin’s lymphoma: A multi-institutional, matched cohort study. J. Clin. Oncol..

[47] Mariscotti G., Durando M., Ghione G., Luparia A., Regini E., Alfieri C., Campanino P.P., Gavarotti P., Brignardello E., Gandini G. (2013). Breast cancer surveillance in patients treated by radiotherapy for Hodgkin’s lymphoma. Radiol. Med..

[48] Ng A.K., Garber J.E., Diller L.R., Birdwell R.L., Feng Y., Neuberg D.S., Silver B., Fisher D.C., Marcus K.J., Mauch P.M. (2013). Prospective study of the efficacy of breast magnetic resonance imaging and mammographic screening in survivors of Hodgkin lymphoma. J. Clin. Oncol..

[49] Andre M., Fortpied C., Viviani S., Bellei M., Carde P., Hutchings M., Gianni A., Brice P., Casasnovas O., Gobbi P. (2016). Overall survival impact of BEACOPP vs ABVD in advanced Hodgkin lymphoma: A pooled analysis of four randomized trials. Haematologica.

[50] Sasse S., Bröckelmann P.J., Goergen H., Plütschow A., Müller H., Kreissl S., Buerkle C., Borchmann S., Fuchs M., Borchmann P. (2017). Long-term follow-up of contemporary treatment in early-stage Hodgkin lymphoma: Updated analysis of the German Hodgkin Study Group HD7, HD8, HD10, and HD11 trials. J. Clin. Oncol..

[51] Engert A., Schiller P., Josting A., Herrmann R., Koch P., Sieber M., Boissevain F., de Wit M., Mezger J., Dühmke E. (2003). Involved-field radiotherapy is equally effective and less toxic compared with extended-field radiotherapy after four cycles of chemotherapy in patients with early-stage unfavorable Hodgkin’s lymphoma: Results of the HD8 trial of the German Hodgkin’s Lymphoma Study Group. J. Clin. Oncol..

[52] Engert A., Plütschow A., Eich H.T., Lohri A., Dörken B., Borchmann P., Berger B., Greil R., Willborn K.C., Wilhelm M. (2010). Reduced treatment intensity in patients with early-stage Hodgkin’s lymphoma. N. Engl. J. Med..

[53] Eich H.T., Diehl V., Görgen H., Pabst T., Markova J., Debus J., Ho A., Dörken B., Rank A., Grosu A.L. (2010). Intensified chemotherapy and dose-reduced involved-field radiotherapy in patients with early unfavorable Hodgkin’s lymphoma: Final analysis of the German Hodgkin Study Group HD11 trial. J. Clin. Oncol..

[54] Frontzek F., Ziepert M., Nickelsen M., Altmann B., Glass B., Haenel M., Truemper L., Held G., Bentz M., Borchmann P. (2019). Conventional chemotherapy (R-CHOEP) vs high-dose immunochemotherapy (R-megaCHOEP) in younger patients with high-risk aggressive B-cell lymphoma: 10-year long-term follow-up of a German Lymphoma Alliance (GLA) Study. Blood.

[55] Bloom J.R., Stewart S.L., Hancock S.L. (2006). Breast Cancer Screening in Women Surviving Hodgkin Disease. Am. J. Clin. Oncol..

[56] Bonadonna G., Zucali R., Monfardini S., De Lena M., Uslenghi C. (1975). Combination chemotherapy of Hodgkin’s disease with adriamycin, bleomycin, vinblastine, and imidazole carboxamide versus MOPP. Cancer.

[57] Valagussa P., Santoro A., Kenda R., Fossati Bellani F., Franchi F., Banfi A., Rilke F., Bonadonna G. (1980). Second malignancies in Hodgkin’s disease: A complication of certain forms of treatment. Br. Med. J..

[58] Brusamolino E., Anselmo A.P., Klersy C., Santoro M., Orlandi E., Pagnucco G., Lunghi F., Maurizi-Enrici R., Baroni C.D., Lazzarino M. (1998). The risk of acute leukemia in patients treated for Hodgkin’s disease is significantly higher after combined modality programs than after chemotherapy alone and is correlated with the extent of radiotherapy and type and duration of chemotherapy: A case-control study. Haematologica.

[59] Canellos G.P., Anderson J.R., Propert K.J., Nissen N., Cooper M.R., Henderson E.S., Green M.R., Gottlieb A., Peterson B.A. (1992). Chemotherapy of advanced Hodgkin’s disease with MOPP, ABVD, or MOPP alternating with ABVD. N. Engl. J. Med..

[60] Casasnovas R.O., Bouabdallah R., Brice P., Lazarovici J., Ghesquieres H., Stamatoullas A., Dupuis J., Gac A.C., Gastinne T., Joly B. (2019). PET-adapted treatment for newly diagnosed advanced Hodgkin lymphoma (AHL2011): A randomized, multicentre, non-inferiority, phase 3 study. Lancet Haematol..

[61] Gallamini A., Tarella C., Viviani S., Rossi A., Patti C., Mulé A., Picardi M., Romano A., Cantonetti M., La Nasa G. (2018). Early chemotherapy intensification with escalated BEACOPP in patients with advanced-stage Hodgkin lymphoma with a positive interim positron emission tomography/computed tomography scan after two ABVD cycles: Long-term results of the GITIL/FIL HD 0607 trial. J. Clin. Oncol..

[62] Hodgson D.C., Koh E.S., Tran T.H., Heydarian M., Tsang R., Pintilie M., Xu T., Huang L., Sachs R.K., Brenner D.J. (2007). Individualized estimates of second cancer risks after contemporary radiation therapy for Hodgkin lymphoma. Cancer.

[63] Koh E.S., Tran T.H., Heydarian M., Sachs R.K., Tsang R.W., Brenner D.J., Pintilie M., Xu T., Chung J., Paul N. (2007). A comparison of mantle versus involved-field radiotherapy for Hodgkin’s lymphoma: Reduction in normal tissue dose and second cancer risk. Radiat. Oncol..

[64] Schneider U., Sumila M., Robotka J., Weber D., Gruber G. (2014). Radiation-induced second malignancies after involved-node radiotherapy with deep-inspiration breath-hold technique for early stage Hodgkin Lymphoma: A dosimetric study. Radiat. Oncol..

[65] Mazonakis M., Lyraraki E., Damilakis J. (2017). Second cancer risk assessments after involved-site radiotherapy for mediastinal Hodgkin lymphoma. Med. Phys..

[66] Campbell B.A., Hornby C., Cunninghame J., Burns M., MacManus M., Ryan G., Lau E., Seymour J.F., Wirth A. (2012). Minimising critical organ irradiation in limited stage Hodgkin lymphoma: A dosimetric study of the benefit of involved node radiotherapy. Ann. Oncol..

[67] Kourinou K.M., Mazonakis M., Lyraraki E., Papadaki H.A., Damilakis J. (2019). Probability of carcinogenesis due to involved field and involved site radiation therapy techniques for supra- and infradiaphragmatic Hodgkin’s disease. Phys. Med..

[68] Aznar M.C., Maraldo M.V., Schut D.A., Lundemann M., Brodin N.P., Vogelius I.R., Berthelsen A.K., Specht L., Petersen P.M. (2015). Minimizing late effects for patients with mediastinal Hodgkin lymphoma: Deep inspiration breath-hold, IMRT, or both?. Int. J. Radiat. Oncol. Biol. Phys..

[69] Maraldo M.V., Specht L. (2014). A decade of comparative dose planning studies for early-stage Hodgkin lymphoma: What can we learn?. Int. J. Radiat. Oncol. Biol. Phys..

[70] Maraldo M.V., Brodin N.P., Aznar M.C., Vogelius I.R., Munck af Rosenschöld P., Petersen P.M., Specht L. (2013). Estimated risk of cardiovascular disease and secondary cancers with modern highly conformal radiotherapy for early-stage mediastinal Hodgkin lymphoma. Ann. Oncol..

[71] Timlin C., Loken J., Kruse J., Miller R., Schneider U. (2021). Comparing second cancer risk for multiple radiotherapy modalities in survivors of hodgkin lymphoma. Br. J. Radiol..

[72] Filippi A.R., Ragona R., Piva C., Scafa D., Fiandra C., Fusella M., Giglioli F.R., Lohr F., Ricardi U. (2015). Optimized volumetric modulated arc therapy versus 3D-CRT for early stage mediastinal Hodgkin lymphoma without axillary involvement: A comparison of second cancers and heart disease risk. Int. J. Radiat. Oncol. Biol. Phys..

[73] Weber D.C., Johanson S., Peguret N., Cozzi L., Olsen D.R. (2011). Predicted risk of radiation-induced cancers after involved field and involved node radiotherapy with or without intensity modulation for early-stage hodgkin lymphoma in female patients. Int. J. Radiat. Oncol. Biol. Phys..

[74] Cella L., Conson M., Pressello M.C., Molinelli S., Schneider U., Donato V., Orecchia R., Salvatore M., Pacelli R. (2013). Hodgkin’s lymphoma emerging radiation treatment techniques: Trade-offs between late radio-induced toxicities and secondary malignant neoplasms. Radiat. Oncol..

[75] Filippi A.R., Ragona R., Fusella M., Botticella A., Fiandra C., Ricardi U. (2013). Changes in breast cancer risk associated with different volumes, doses, and techniques in female Hodgkin lymphoma patients treated with supra-diaphragmatic radiation therapy. Pract. Radiat. Oncol..

[76] Hill D.A., Gilbert E., Dores G.M., Gospodarowicz M., van Leeuwen F.E., Holowaty E., Glimelius B., Andersson M., Wiklund T., Lynch C.F. (2005). Breast cancer risk following radiotherapy for Hodgkin lymphoma: Modification by other risk factors. Blood.

[77] Filippi A.R., Meregalli S., DIRusso A., Levis M., Ciammella P., Buglione M., Guerini A.E., De Marco G., De Sanctis V., Vagge S. (2020). Fondazione Italiana Linfomi (FIL) Radiotherapy Committee. Fondazione Italiana Linfomi (FIL) expert consensus on the use of intensity-modulated and image-guided radiotherapy for Hodgkin’s lymphoma involving the mediastinum. Radiat. Oncol..

[78] Manem V.S.K., Dhawan A. (2018). Modelling recurrence and second cancer risks induced by proton therapy. Math. Med. Biol..

[79] Horn S., Fournier-Bidoz N., Pernin V., Peurien D., Vaillant M., Dendale R., Fourquet A., Kirova Y.M. (2016). Comparison of passive-beam proton therapy, helical tomotherapy and 3D conformal radiation therapy in Hodgkin’s lymphoma female patients receiving involved-field or involved site radiation therapy. Cancer Radiother..

[80] Hodgson D.C., Gilbert E.S., Dores G.M., Schonfeld S.J., Lynch C.F., Storm H., Hall P., Langmark F., Pukkala E., Andersson M. (2007). Long-Term Solid Cancer Risk Among 5-Year Survivors of Hodgkin’s Lymphoma. J. Clin. Oncol..

[81] Dores G.M., Metayer C., Curtis R.E., Lynch C.F., Clarke E.A., Glimelius B., Storm H., Pukkala E., van Leeuwen F.E., Holowaty E.J. (2002). Second malignant neoplasms among long-term survivors of Hodgkin’s disease: A population-based evaluation over 25 years. J. Clin. Oncol..

[82] Bhatia S., Yasui Y., Robison L.L., Birch J.M., Bogue M.K., Diller L., DeLaat C., Fossati-Bellani F., Morgan E., Oberlin O. (2003). Late Effects Study Group. High risk of subsequent neoplasms continues with extended follow-up of childhood Hodgkin’s disease: Report from the Late Effects Study Group. J. Clin. Oncol..

[83] Bröckelmann P.J., Eichenauer D.A., Jakob T., Follmann M., Engert A., Skoetz N. (2018). Hodgkin Lymphoma in Adults. Dtsch. Arztebl. Int..

[84] Ha C.S., Hodgson D.C., Advani R., Dabaja B.S., Dhakal S., Flowers C.R., Hoppe B.S., Mendenhall N.P., Metzger M.L., Plastaras J.P. (2014). ACR appropriateness criteria follow-up of Hodgkin lymphoma. J. Am. Coll. Radiol..

[85] Hodgson D.C. (2008). Hodgkin lymphoma: The follow-up of long-term survivors. Hematol. Oncol. Clin. N. Am..

[86] Hodgson D.C., Grunfeld E., Gunraj N., Del Giudice L. (2010). A population-based study of follow-up care for Hodgkin lymphoma survivors: Opportunities to improve surveillance for relapse and late effects. Cancer.

[87] Travis L.B., Gospodarowicz M., Curtis R.E., Clarke E.A., Andersson M., Glimelius B., Joensuu T., Lynch C.F., van Leeuwen F.E., Holowaty E. (2002). Lung cancer following chemotherapy and radiotherapy for Hodgkin’s disease. J. Natl. Cancer Inst..

[88] Das P., Ng A.K., Earle C.C., Mauch P.M., Kuntz K.M. (2006). Computed tomography screening for lung cancer in Hodgkin’s lymphoma survivors: Decision analysis and cost-effectiveness analysis. Ann. Oncol..

[89] Wattson D.A., Hunink M.G., DiPiro P.J., Das P., Hodgson D.C., Mauch P.M., Ng A.K. (2014). Low-dose chest computed tomography for lung cancer screening among Hodgkin lymphoma survivors: A cost-effectiveness analysis. Int. J. Radiat. Oncol. Biol. Phys..

[90] (2021). NCCN Clinical Practice Guidelines in Oncology. https://www.nccn.org/professionals/physician_gls/pdf/survivorship.pdf.

[91] Curry S.J., Krist A.H., Owens D.K., Barry M.J., Caughey A.B., Davidson K.W., Doubeni C.A., Epling J.W., Kemper A.R., Kubik M. (2018). US Preventive Services Task Force. Screening for cervical cancer: US Preventive Services Task Force recommendation statement. JAMA.

