# Supplementary material for: Second Cancers in Classical Hodgkin Lymphoma and Diffuse Large B-Cell Lymphoma: A Systematic Review by the Fondazione Italiana Linfomi
Source: Cancers (Basel). 2022 Jan 20;14(3):519. doi: 10.3390/cancers14030519 (PMC8833346; doi:10.3390/cancers14030519)
Supplement: Supplementary file 1 [file cancers-14-00519-s001.zip › Data S1_search strategies.pdf]

Supplementary Materials: Second Cancers in Classical Hodgkin Lymphoma and Diffuse Large B-Cell Lymphoma. A Systematic Review by the Fondazione Italiana Linfomi

Luca Nassi, Vitaliana De Sanctis, Giacomo Loseto, Chiara Gerardi, Eleonora Allocati, Sabino Ciavarella, Carla Minoia, Attilio Guarini and Alessia Bari

PICO A_INCIDENCE

EMBASE

***N* = 66**

('hodgkin disease'/exp OR 'hodgkin disease' OR 'diffuse large b cell lymphoma')

AND

('consolidation chemotherapy' OR 'induction chemotherapy' OR 'conventional chemotherapy' OR 'chemotherapy' OR 'chemotherapy'/exp OR 'cyclophosphamide plus doxorubicin plus prednisolone plus rituximab plus vincristine' OR 'cyclophosphamide doxorubicin vincristine prednisone' OR 'chop protocol' OR 'abvd protocol' OR 'high dose protocol' OR 'high dose chemotherapy' OR 'autologous stem cell transplantation' OR 'transplantation' OR 'hematopoietic stem cell graft' OR 'hematopoietic stem cell transplantation' OR 'brentuximab' OR 'antineoplastic agent' OR 'antineoplastic agent'/exp OR 'antineoplastic protocol' OR 'adjuvant chemotherapy' OR 'chemoradiotherapy' OR 'adjuvant chemoradiotherapy')

AND

('long term survival' OR 'long term survival'/exp)

AND

('second cancer' OR 'second cancer'/exp OR 'second tumor' OR 'neoplasm radiotherapy' OR 'radiation induced neoplasm' OR 'radiation induced neoplasm'/exp OR 'lymphoma relapse' OR 'lymphoma recurrence')

COCHRANE

***N* = 8**

ID Search

#1 Hodgkin's disease

#2 MeSH descriptor: [Hodgkin Disease] explode all trees

#3 Hodgkin's lymphoma

#4 B-cell lymphoma

#5 MeSH descriptor: [Lymphoma, B-Cell] explode all trees

#6 #1 OR #2 OR #3 OR #4 OR #5

#7 "consolidation chemotherapy" OR "induction chemotherapy" OR "conventional chemotherapy" OR "chemotherapy" OR "cyclophosphamide plus doxorubicin plus prednisolone plus rituximab plus vincristine" OR "cyclophosphamide doxorubicin vincristine prednisone" OR "chop protocol" OR "abvd protocol" OR "high dose protocol" OR "high dose chemotherapy" OR "autologous stem cell transplantation" OR "transplantation" OR "hematopoietic stem cell graft" OR "hematopoietic stem cell transplantation' OR 'brentuximab" OR "antineoplastic agent" OR "antineoplastic protocol" OR "adjuvant chemotherapy" OR "chemoradiotherapy" OR "adjuvant chemoradiotherapy"

#8 MeSH descriptor: [Drug Therapy] explode all trees

#9 MeSH descriptor: [Antineoplastic Agents] this term only

#10 #7 OR #8 OR #9
#11 'long term surv*'
#12 MeSH descriptor: [Cancer Survivors] explode all trees
#13 #11 OR #12
#14 "second cancer" OR "second tumor" OR "neoplasm radiotherapy" OR "radiation induced neoplasm" OR "lymphoma relapse" OR "lymphoma recurrence"
#15 MeSH descriptor: [Neoplasms, Second Primary] explode all trees
#16 MeSH descriptor: [Neoplasms, Radiation-Induced] explode all trees
#17 #14 OR #15 OR #16
#18 #6 AND #10 AND #13 AND #17
#19 "accession number" near pubmed
#20 "accession number" near EMBASE
#21 #19 OR #20
#22 #18 NOT #21

PICO B_ COMPARISON OF THERAPIES
EMBASE
***N* = 56**
('hodgkin disease'/exp OR 'hodgkin disease' OR 'diffuse large b cell lymphoma')
AND
('second cancer' OR 'second cancer'/exp OR 'second tumor' OR 'neoplasm radiotherapy' OR 'radiation induced neoplasm' OR 'radiation induced neoplasm'/exp OR 'lymphoma relapse' OR 'lymphoma recurrence')
AND
('radiotherapy' OR 'radiotherapy'/exp OR 'radiotherapy planning system' OR 'radiotherapy planning system'/exp OR 'conformal radiotherapy' OR 'involved field radiotherapy' OR 'mantle field radiotherapy'/exp OR 'involved site radiation therapy' OR 'involved nodal radiotherapy' OR 'proton therapy' OR 'image guided radiotherapy' OR 'intensity modulated radiation therapy')
AND
('long term survival' OR 'long term survival'/exp)

COCHRANE
***N* = 6**
ID Search
#1 Hodgkin's disease
#2 MeSH descriptor: [Hodgkin Disease] explode all trees
#3 Hodgkin's lymphoma
#4 B-cell lymphoma
#5 MeSH descriptor: [Lymphoma, B-Cell] explode all trees
#6 #1 OR #2 OR #3 OR #4 OR #5
#7 'long term surv*'
#8 MeSH descriptor: [Cancer Survivors] explode all trees
#9 #7 OR #8

#10 "second cancer" OR "second tumor" OR "neoplasm radiotherapy" OR "radiation induced neoplasm" OR "lymphoma relapse" OR "lymphoma recurrence"
#11 MeSH descriptor: [Neoplasms, Second Primary] explode all trees
#12 MeSH descriptor: [Neoplasms, Radiation-Induced] explode all trees
#13 #10 OR #11 OR #12
#14 radiotherapy
#15 MeSH descriptor: [Radiotherapy] explode all trees
#16 "radiotherapy planning system"
#17 "conformal radiotherapy"
#18 MeSH descriptor: [Radiotherapy, Conformal] explode all trees
#19 "involved field radiotherapy"
#20 "mantle field radiotherapy"
#21 "involved site radiation therapy"
#22 "proton therapy"
#23 "image guided radiotherapy"
#24 "intensity modulated radiation therapy"
#25 #14 OR #15 OR #16 OR #17 OR #18 OR #19 OR #20 OR #21 OR #22 OR #23 OR #24
#26 #6 AND #9 AND #13 AND #25
#27 "accession number" near pubmed
#28 "accession number" near EMBASE
#29 #27 OR #28
#30 #26 NOT #29

PICO C_FOLLOW UP
EMBASE 15/01/2020
***N* = 650**
('hodgkin disease'/exp OR 'hodgkin disease' OR 'diffuse large b cell lymphoma')
AND
('consolidation chemotherapy' OR 'induction chemotherapy' OR 'conventional chemotherapy' OR 'chemotherapy' OR 'chemotherapy'/exp OR 'cyclophosphamide plus doxorubicin plus prednisolone plus rituximab plus vincristine' OR 'cyclophosphamide doxorubicin vincristine prednisone' OR 'chop protocol' OR 'abvd protocol' OR 'high dose protocol' OR 'high dose chemotherapy' OR 'autologous stem cell transplantation' OR 'transplantation' OR 'hematopoietic stem cell graft' OR 'hematopoietic stem cell transplantation' OR 'brentuximab' OR 'antineoplastic agent' OR 'antineoplastic agent'/exp OR 'antineoplastic protocol' OR 'adjuvant chemotherapy' OR 'chemoradiotherapy' OR 'adjuvant chemoradiotherapy')
AND
('late side effect' OR 'adverse event' OR 'late onset' OR 'adverse event'/exp OR 'late diagnosis' OR 'long term care' OR 'long term care'/exp OR 'patient care' OR 'patient care planning' OR 'patient care'/exp OR 'surveillance' OR 'health care planning' OR 'health care planning'/exp)
AND
('second cancer' OR 'second cancer'/exp OR 'second tumor' OR 'neoplasm radiotherapy' OR 'radiation induced neoplasm' OR 'radiation induced neoplasm'/exp OR 'lymphoma relapse' OR 'lymphoma recurrence')
AND [embase]/lim

COCHRANE

***N* = 6**

ID Search

#1 Hodgkin's disease

#2 MeSH descriptor: [Hodgkin Disease] explode all trees

#3 Hodgkin's lymphoma

#4 B-cell lymphoma

#5 MeSH descriptor: [Lymphoma, B-Cell] explode all trees

#6 #1 OR #2 OR #3 OR #4 OR #5

#7 "consolidation chemotherapy" OR "induction chemotherapy" OR "conventional chemotherapy" OR "chemotherapy" OR "cyclophosphamide plus doxorubicin plus prednisolone plus rituximab plus vincristine" OR "cyclophosphamide doxorubicin vincristine prednisone" OR "chop protocol" OR "abvd protocol" OR "high dose protocol" OR "high dose chemotherapy" OR "autologous stem cell transplantation" OR "transplantation" OR "hematopoietic stem cell graft" OR "hematopoietic stem cell transplantation' OR 'brentuximab" OR "antineoplastic agent" OR "antineoplastic protocol" OR "adjuvant chemotherapy" OR "chemoradiotherapy" OR "adjuvant chemoradiotherapy"

#8 MeSH descriptor: [Drug Therapy] explode all trees

#9 MeSH descriptor: [Antineoplastic Agents] this term only

#10 #7 OR #8 OR #9

#11 "late side effect" OR "adverse event" OR "late onset" OR "late diagnosis" OR "long term care" OR "patient care" OR "patient care planning" OR "surveillance" OR "health care planning"

#12 MeSH descriptor: [Long-Term Care] explode all trees

#13 MeSH descriptor: [Patient Care] explode all trees

#14 #11 OR #12 OR #13

#15 "second cancer" OR "second tumor" OR "neoplasm radiotherapy" OR "radiation induced neoplasm" OR "lymphoma relapse" OR "lymphoma recurrence"

#16 MeSH descriptor: [Neoplasms, Second Primary] explode all trees

#17 MeSH descriptor: [Neoplasms, Radiation-Induced] explode all trees

#18 #15 OR #16 OR #17

#19 #6 AND #10 AND #14 AND #18

#20 "accession number" near pubmed

#21 "accession number" near EMBASE

#22 #20 OR #21

#23 #19 NOT #22
